# Neuroanatomical associations with autistic characteristics in those with acute anorexia nervosa and weight-restored individuals

**DOI:** 10.1017/S0033291725001047

**Published:** 2025-04-28

**Authors:** Michelle Sader, Daniel Halls, Jess Kerr-Gaffney, Gordon D. Waiter, Karri Gillespie-Smith, Fiona Duffy, Kate Tchanturia

**Affiliations:** 1 School of Medicine, Medical Sciences and Nutrition, University of Aberdeen, Aberdeen, Scotland, UK; 2 Eating Disorders and Autism Collaborative (EDAC), University of Edinburgh, Edinburgh, Scotland, UK; 3 Department of Psychological Medicine, King’s College London, UK; 4 School of Health in Social Science, University of Edinburgh, Edinburgh, Scotland, UK; 5 NHS Lothian Child and Adolescent Mental Health Services, Royal Edinburgh Hospital, Edinburgh, Scotland, UK; 6 Department of Psychology, Illia State University, Tbilisi, Georgia

**Keywords:** anorexia nervosa, autism, body mass index, magnetic resonance imaging

## Abstract

Common neuroanatomical regions are associated with both states of anorexia nervosa (AN) and autistic characteristics, but restoration of body mass index (BMI) has been associated with decreased presentation of autistic characteristics in some individuals with AN. This study aims to examine neuroanatomical correlates associated with autistic characteristics in those with acute anorexia nervosa (ac-AN) and those previously diagnosed with AN but whose weight has been restored (WR). In total, 183 individuals (healthy controls [HCs] = 67; n[ac-AN] = 68; n[WR] = 48) from the Brain imaging of Emotion And Cognition of adolescents with Anorexia Nervosa (BEACON) study were included, with autistic characteristics determined in both ac-AN and WR individuals (n = 116). To further examine BMI, ac-AN and WR group associations were compared. Random forest regression (RFR) models examined whether autistic characteristics and morphology of the anterior cingulate cortex (ACC), middle frontal gyrus (MFG), and orbitofrontal cortex (OFC) were able to predict future levels of social anhedonia and alexithymia. Group-wise differences were identified within the volume and surface area of the MFG and OFC, which were unrelated to BMI. Autistic characteristics were inversely associated with MFG and ACC volume, with differences in associations between ac-AN and WR groups seen in the surface area of the MFG. RFR models identified moderate-to-weak performance and found that autistic characteristics were not important predictive features in a priori and exploratory models. Findings suggest that the presence of autistic characteristics in those with ac-AN are associated with the volume of the MFG and are unrelated to BMI restoration.

## Introduction

Anorexia nervosa (AN) is a severe and life-threatening eating disorder (ED) characterized by pathological fears of weight gain, a distorted body image, and extremely low body weight in relation to individual age and sex (American Psychiatric Association, 2013). Those with AN exhibit significantly elevated levels of autistic traits ranging between 2% and 53% (Leppanen, Sedgewick, Halls, & Tchanturia, 2022; Nickel et al., 2019; Westwood & Tchanturia, 2017) and share behavioral characteristics (Kerr-Gaffney et al., 2021; Westwood & Tchanturia, 2017). Autism is a neurodevelopmental condition and presents as a distinct neurotype or way with which the brain processes information, characterized by a wide range of divergences in cognition and methods of communication (American Psychiatric Association, 2013). AN and autism share behavioral features such as expression of special interests and repetitive behaviors as well as differences relative to non-Autistic individuals without AN in social communication, theory of mind, and central coherence (Kerr-Gaffney et al., 2021; Westwood, Stahl, Mandy, & Tchanturia, 2016), which are also associated with prolonged AN illness course (Saure et al., 2020). Overlaps between AN states and autistic characteristics have been reported to partially diminish upon ED recovery (Susanin et al., 2022). However, recent studies found no relationship between BMI and presentation of autistic characteristics (Kerr-Gaffney et al., 2021; Nuyttens, Simons, Antrop, & Glazemakers, 2024). Due to a lack of consensus on this relationship, many individuals with acute anorexia nervosa (ac-AN) do not receive autism assessments due to being classified as ‘acutely ill’, and autistic characteristics may be secondary to states of starvation (Kerr-Gaffney et al., 2021; Treasure, 2013). This has the potential for health services to overlook autistic individuals with ac-AN and contributes to limited understanding concerning which phenotypes are associated with AN relative to being autistic-driven.

An important consideration clouding current understanding of the overlap between AN states and autistic characteristics is how recovery from AN is conceptualized and defined. Multiple studies utilize body mass index (BMI) as a key criterion necessary to define recovery, while other studies incorporate the presence (or absence) of psychiatric symptoms over a set time period (Khalsa, Portnoff, McCurdy-McKinnon, & Feusner, 2017). AN recovery exhibits more nuance than weight restoration, which overlooks multiple psychological and emotional factors associated with AN (Barko & Moorman, 2023). Inconsistencies across diagnostic definitions raise the question as to how remission, or temporary resolution of symptoms, differs from ‘true’ AN recovery (Bardone-Cone, Hunt, & Watson, 2018), including how relationships between the presence of AN symptomatology and autistic characteristics play a role in this debate. The Brain imaging of Emotion And Cognition of adolescents with Anorexia Nervosa (BEACON) study (Halls et al., 2021) is a longitudinal neuroimaging project evaluating socioemotional and structural/functional brain differences in those with ac-AN relative to healthy controls (HCs) and weight-restored (WR) individuals. Given the importance of investigating the overlap between autistic characteristics and AN while distinguishing between ac-AN and WR individuals, the BEACON study provides data to address this gap.

Structural magnetic resonance imaging (MRI) research reports similarities in brain structure between those with AN and autistic individuals, reporting differential structure of the amygdala (Schumann, Bauman, & Amaral, 2011; Wronski et al., 2023), cerebellum (Fonville et al., 2014; Sydnor & Aldinger, 2022), insula (Alfano, Mele, Cotugno, & Longarzo, 2020; Doyle-Thomas et al., 2013), cingulate cortex (Alfano, Mele, Cotugno, & Longarzo, 2020; Doyle-Thomas et al., 2013; Sader, Williams, & Waiter, 2022), as well as orbitofrontal and frontal cortex (Alfano, Mele, Cotugno, & Longarzo, 2020; Richey et al., 2022; Sader, Williams, & Waiter, 2022; Zielinski et al., 2014) in both groups relative to their respective nonautistic and HC non-AN comparators. Shared differences include neuroanatomical parameters essential for brain development (Vijayakumar et al., 2016), such as cortical thickness and surface area (Ecker et al., 2013; Lavagnino et al., 2018; Leppanen et al., 2019; Mishima et al., 2021; Nickel et al., 2018). Evidence also suggests limited neuroanatomical overlap between those with AN and autistic individuals (Halls et al., 2022; Sader, Williams, & Waiter, 2022). A previous meta-analysis examining the overlap between both groups identified the anterior cingulate cortex (ACC), orbitofrontal cortex (OFC), and middle frontal gyrus (MFG) as associated with AN versus non-AN HCs, but no differences were identified in autistic versus nonautistic groups (Sader, Williams, & Waiter, 2022). Further research is necessary to disentangle the complex relationship between autistic characteristics and AN-related symptomatology.

This study aims to examine the (1) structural associations between autistic characteristics and brain structure in AN; (2) alteration of existing neuroanatomical correlations according to BMI; (3) prediction of future socioemotional and cognitive behavioral characteristics based on volumetric structure and strength of autistic characteristics. It was hypothesized that (1) structural associations with autistic characteristics will be observed within the OFC, ACC, and MFG in those with AN, confirming previous reports (Sader, Williams, & Waiter, 2022); (2) existing associations will not differ across BMI; (3) associations between OFC, ACC, and MFG structure and strength of autistic characteristics will successfully predict future levels of alexithymia and social anhedonia.

## Materials and methods

### Participant characteristics

The study sample was derived from BEACON, containing individuals with sociodemographic, socioemotional, and cognitive behavior measurements, as well as data on brain structure and function. From N = 190 (nAN = 117 [nac-AN = 58; nWR = 60]; nHC = 70) sample, n = 6 were excluded due to poor scan quality, and n = 1 due to missing sociodemographic data for a final sample of N = 183 (nAN = 116 [nac-AN = 68; nWR = 48]; nHC = 67). Participants were assessed at two time points, with time point 1 (TP1) occurring between June 2017 and February 2019 and time point 2 (TP2) occurring between October 2019 and March 2022, with a mean follow-up time of 2.61 years ±6.67 months.

Participant eligibility included those who were right-handed, aged between 12 and 27 years, had no history of brain injury, intellectual disability or neurological impairment, and could undergo scanning procedures. Individuals with ac-AN were classified based on existing AN DSM-5 criteria, stating individuals should fall below a BMI of <18.5 if 18+ years or < 85th median BMI percentile in those <18 years (Halls et al., 2021). WR individuals were characterized as having a previous AN diagnosis but falling within a healthy BMI range (18.5–25) if 18+ years, or > 85th median BMI percentile if <18 years. HCs contained no ED diagnosis history and fell within a BMI of 18.5–25. Participants provided written-informed consent, with the study approved by the National Research Ethics Committee (17/LO/2071). All research was performed in accordance with the Declaration of Helsinki (World Medical Association, 2013). Further information on demographic characteristics or recruitment may be found within the *Supplementary Material* (Materials & Methods) and Halls et al. (2021).

### Clinical and self-report measures

The 36-item self-report Eating Disorder Examination Questionnaire (EDE-Q 6.0; Fairburn & Beglin, 2008) was used to evaluate ED features. To evaluate autistic characteristics, the 10-item Autism Quotient (AQ-10; Allison, Auyeung, & Baron-Cohen, 2012), a brief self-report screening tool to determine the presence of autistic characteristics and widely used in ED research (Westwood et al., 2016), was used. Participants also completed the Autism Diagnostic Observation Schedule, 2nd edition (ADOS-2; Lord et al., 2012), a standardized diagnostic assessment of autism evaluating communication, social interaction, and restricted and/or repetitive behaviors. Due to significant presence of missing-at-random values, mean AQ-10 scores were used for analysis to answer research questions.

Additional collected measures focused on a range of psychological behaviors and symptoms. The 14-item self-report Hospital Anxiety and Depression Scale (HADS; Zigmond & Snaith, 1983) was used to evaluate levels of anxiety and depression. The 18-item self-report Obsessive-Compulsive Inventory-Revised (OCI-R; Foa et al., 2002) was used to measure levels of obsessive-compulsive disorder (OCD). Levels of social anhedonia (pleasure derived from social experiences) were assessed using the 40-item self-report Social Anhedonia Scale (SAS; Eckblad, Chapman, Chapman, & Mishlove, 1982). The level of general functioning due to illness was examined using the brief 5-item self-report Work and Social Adjustment Scale (WSAS; Mundt, Marks, Shear, & Greist, 2002). The presence of alexithymia or the ability to discern emotional states was investigated via the 20-item Toronto Alexithymia Scale (TAS-20; Bagby, Parker, & Taylor, 1994). Across self-report measures, internal consistency via Cronbach’s alpha (α) ranged from acceptable to excellent (EDE-Q = 0.97 [0.96,0.97]; AQ-10 = 0.75 [0.69,0.80]; HADS = 0.90 [0.87,0.92]; OCI-R = 0.94 [0.92,0.95]; SAS = 0.91 [0.88, 0.93]; WSAS = 0.93 [0.91, 0.95]; TAS-20 = 0.90 [0.87, 0.93]). The intelligence quotient (IQ) of participants was evaluated using the National Adult Reading Test (NART) (Nelson & Willison, 1991). Demographic questionnaires collected data on BMI (height/weight^2^), age, and duration-of-illness (DOI). Missing time-of-scan BMI values were univariately imputed from baseline BMI using the ‘mice()’ R package.

### Scanning parameters and MRI data

Scans were acquired on a 3 T GE MRI scanner, located within the Centre for Neuroimaging Sciences at King’s College London. Structural T1-weighted images collected prior to functional image acquisition were used for analysis with the following parameters consisting of an echo and repetition time of 3.016 s and 7.312 s and a field of view of 270 mm with a slice thickness of 1.2 mm. The flip angle for scans was set to 11° with a matrix of 256 × 256 pixels. Participants spent approximately 30 minutes in the scan. The ENIGMA Cortical Quality Control Protocol 2.0 (University of Southern California, 2017) was used to examine the pre-processed output from FreeSurfer for quality control issues. All images were preprocessed and analyzed using FreeSurfer (Version 6.0; https://surfer.nmr.mgh.harvard.edu/). Global and regional values for volume, surface area, and cortical thickness were extracted from preprocessed images following the Desikan–Killiany atlas. For further information on MRI parameters, see the *Supplementary Material* (Materials & Methods) and Halls et al. (2021).

### Statistical analysis

Statistical tests were performed using the R Software (Version 4.2.1.). As the aim of this study is to evaluate neural correlates in ac-AN relative to WR individuals, primary analyses assessed ac-AN versus WR groups, with additional provided analyses evaluating ac-AN versus HC groups. Data with non-normal distribution patterns were log transformed and assessed via parametric means using 2-sample t-tests under a p < 0.05 threshold adjusted for multiple comparisons using the Bonferroni correction. Non-normal distributions post-log transformations used the ‘bestNormalize()’ R package. For data with non-normal distributions post-transformation, Mann–Whitney *U* tests assessed group differences. To ease meaningful interpretation of results, Bayes factor (BF) bounds were calculated for all tests from p values using the ‘piercer()’ R package. BF bounds were reported between values of <1.60 and >53.00, corresponding to p values of p > 0.1–p < 0.001, respectively (Benjamin & Berger, 2019). A priori analyses assessed whether brain morphometry in the ACC, MFG, and OFC differed between ac-AN versus WR groups and between ac-AN versus HC groups. Excluding cortical thickness values, brain volume was corrected for head size using the proportional method. To investigate regions not identified via a priori analysis, an agnostic/whole-brain approach was also performed. For agnostic analyses, results were corrected for multiple comparisons via the Bonferroni correction. Across assessments, age, NART, DOI, and use of medication (yes/no) were included as covariates. NART scores were included as previous studies report high IQ in AN relative to HCs (Lopez, Stahl, & Tchanturia, 2010; Schilder et al., 2017). DOI (in years), determined according to group-based BMI classifications, was included as previous literature reports associations between the volume of regions such as the insula or cerebellum and AN DOI (Fonville et al., 2014; Zucker et al., 2017).

To assess associations between brain structure and autistic characteristics in those with AN, multiple linear regressions evaluated structural brain parameters alongside AQ-10 scores. To explore whether associations between brain structure and autistic characteristics are influenced by BMI, three-path mediation analyses were conducted using the ‘mediation()’ package, with the predictor variable serving as structural brain parameters and the outcome variable serving as AQ-10 scores, with BMI as the mediation variable. To assess associations between neuroanatomical parameters and autistic characteristics in AN, HCs were excluded from analysis (n = 116), with the ac-AN (n = 68) and WR (n = 48) groups used and differences in associations were compared using Fisher r-to-z transforms. Age, NART, DOI, and use of medication were included as covariates for all analyses.

To determine whether a priori volumetric structure alongside strength of autistic characteristics can predict future levels of alexithymia and social anhedonia, a random forest regression (RFR) approach was used using the ‘randomForest()’ R package. RFRs follow an ensemble learning method utilizing multiple random decision trees trained from subsets of data. RFRs have been used with high accuracy in the classification of other disorders such as depression (Zhou et al., 2024) and Alzheimer’s disease (Sarica, Cerasa, & Quattrone, 2017). RFR is reported to perform well across varying sample sizes and produce accurate results despite the risks of overfitting, the use of highly non-linear data, and its lack of susceptibility to noise (Sarica, Cerasa, & Quattrone, 2017). RFR models predicted future levels of social anhedonia and alexithymia using the SAS and TAS-20, respectively. Model accuracy and performance were reported using out-of-bag (OOB) error, a measurement of model prediction error when performed on the tested dataset, mean squared error (MSE), a measurement of the average squared difference between actual relative to predicted values and R^2^ values. Further information on RFR model development may be found in the *Supplementary Materials* (Statistical Analysis).

## Results

### Sociodemographic characteristics

Relative to HC individuals, those with ac-AN had significantly lower BMI (t = −13.52; p[Bonferroni] < 5.00E−15), and significantly higher EDE-Q scores (t = 8.36; p[Bonferroni] = 3.20E−12), including the restraint (t = 7.67; p[Bonferroni] = 1.25E−12), eating concern (t = 9.27; p[Bonferroni] = 2.34E−14), shape concern (t = 7.81; p[Bonferroni] = 6.00E−11) and weight concern (t = 7.54; p[Bonferroni] = 2.50E−10) subscales. Those with ac-AN also exhibited higher scores on the ADOS-2 (t = 3.45; p[Bonferroni] = 0.019), AQ-10 (t = 4.05; p[Bonferroni] = 0.0023), HADS (t = 7.83; p[Bonferroni] = 5.55E−11), OCI-R (t = 5.26; p[Bonferroni]1.62E−05), and WSAS (t = 8.87; p[Bonferroni] = 2.05E−13) scores. Relative to HCs, those with ac-AN exhibited a higher prevalence of medication use (X^2^ = 16.04; p[Bonferroni] = 0.0016), particularly antidepressants (X^2^ = 18.15; p[Bonferroni] = 5.10E−04). Relative to WR individuals, those with ac-AN demonstrated significantly lower BMI (t = −10.44; p[Bonferroni] < 5.00E−15) (Table 1).

**Table 1.** Sociodemographic characteristics according to HC (n = 67), WR (n = 48) and ac-AN (n = 68) BMI group

Characteristics		Total (N = 183)	HC (n = 67)	WR (n = 48)	ac-AN (n = 68)	t^a^	t^b^	p^a^/BF^a^	p^b^/BF^b^	p(Bon)^a^ /BF^a^	p(Bon)^b^ /BF^b^
**Age (Y)**^†^		19.05 ± 3.29	19.45 ± 3.38	18.92 ± 3.51	18.75 ± 3.05	**2.07**	−0.14	**0.041/2.82**	0.89/<1.60	1.00/<1.60	1.00/<1.60
Race (N, %)	White	151, 82.51%	54, 80.60%	44, 91.67%	53, 77.94%	X^2^ = 0.028	X^2^ = 2.93	0.87/<1.60	0.087/1.73	1.00/<1.60	0.348/<1.60
	Black	9, 4.92%	5, 7.46%	0, 0.00%	4, 5.88%	X^2^ = 0.00053	X^2^ = 1.42	0.98/<1.60	0.23/<1.60	1.00/<1.60	0.920/<1.60
	Asian/South Asian	7, 3.83%	2, 2.99%	1, 2.083%	4, 5.88%	X^2^ = 0.16	X^2^ = 0.28	0.69/<1.60	0.60/<1.60	1.00/<1.60	1.00/<1.60
	Multi-racial	12, 6.56%	5, 7.46%	2, 4.17%	5, 7.35%	X^2^ = 1.38E–31	X^2^ = 0.099	1.00/<1.60	0.75/<1.60	1.00/<1.60	1.00/<1.60
**BMI**		19.97 ± 3.64^1^; 2.96 ± 0.97^2^	22.73 ± 3.30^1^; 3.10 ± 0.60^2^	20.88 ± 2.25^1^; 3.00 ± 0.37^2^	16.60 ± 1.44^1^; 2.82 ± 0.38^2^	**13.52**	**10.44**	**<2.00E–16** **/>53.00**	**<2.00E–16** **/>53.00**	**<5.00E–15** **/>53.00**	**<5.00e–15** **/>53.00**
**IQ**^†^		110.66 ± 7.51	109.0 ± 6.95	111.60 ± 7.57	111.70 ± 7.81	**−2.05**	−1.31	**0.043/2.73**	0.20/<1.60	1.00/<1.60	1.00/<1.60
Illness duration (Y)^†^		3.72 ± 2.76	N/A	4.26 ± 2.80	3.37 ± 2.70	N/A	1.81	N/A/N/A	0.063/2.12	N/A/N/A	1.00/<1.60
**EDE-Q total score**^†^		2.17 ± 1.83	0.58 ± 0.83	2.80 ± 1.63	3.28 ± 1.57	**−8.36**	−1.34	**1.28E–13/>53.00**	0.18/<1.60	**3.20E–12/>53.00**	1.00/<1.60
**EDE-Q restraint score**^†^		1.96 ± 1.88	0.46 ± 0.81	2.52 ± 1.82	3.06 ± 1.72	**−7.67**	−1.62	**5.00E–12/>53.00**	0.11/<1.60	**1.25E–10/>53.00**	1.00/<1.60
**EDE-Q eating concern score**^†^		1.68 ± 1.71	0.20 ± 0.62	2.17 ± 1.54	2.78 ± 1.53	**−9.27**	**−2.17**	**9.34E–16/>53.00**	**0.033/3.30**	**2.34E–14/>53.00**	0.82/<1.60
**EDE-Q shape concern score**^†^		2.75 ± 2.10	0.94 ± 1.15	3.54 ± 1.86	3.97 ± 1.74	**−7.81**	−1.03	**2.40E–12/>53.00**	0.30/<1.60	**6.00E–11/>53.00**	1.00/<1.60
**EDE-Q weight concern score**^†^		2.28 ± 1.97	0.74 ± 1.06	2.99 ± 1.88	3.29 ± 1.78	**−7.54**	−0.76	**9.98E–12/>53.00**	0.45/<1.60	**2.50E–10/>53.00**	1.00/<1.60
**% Meeting EDE-Q Criteria (N, %)**^‡^		37; 20.22%	1; 1.49%	12; 25.00%	24; 35.29%	**X**^**2**^ **= 23.36**	X^2^ = 0.95	**1.34E–06/>53.00**	0.33/<1.60	**3.35E–05/>53.00**	1.00/<1.60
**ADOS–2 comm. score**^†^		1.29 ± 1.47	0.68 ± 1.08	1.36 ± 1.56	1.84 ± 1.52	**−3.19**	**−2.09**	**0.0018/31.75**	**0.040/2.85**	**0.046/2.60**	1.00/<1.60
**ADOS–2 Comm. + SI score**^†^		2.77 ± 2.75	1.59 ± 2.09	2.70 ± 2.96	4.03 ± 2.65	**−3.21**	**−2.47**	**0.0017/34.27**	**0.016/5.70**	**0.042/2.76**	0.39/<1.60
**ADOS–2 total score**^†^		7.16 ± 4.92	4.76 ± 3.40	7.49 ± 5.29	9.33 ± 4.90	**−3.45**	**−2.12**	**7.69E–04/>53.00**	**0.037/3.03**	**0.019/4.85**	0.92/<1.60
% Meeting ADOS–2 criteria (N, %)^‡^		12; 6.56%	2; 2.99%	2; 4.17%	8; 11.76%	X^2^ = 2.62	X^2^ = 1.21	0.11/<1.60	0.27/<1.60	1.00/<1.60	1.00/<1.60
**% medication**^‡^		71; 38.80%	10; 14.93%	28; 53.83%	33; 48.53%	**X**^**2**^ **= 16.04**	X^2^ = 0.73	**6.19E–05/>53.00**	0.39/<1.60	**0.0016/3.67**	1.00/<1.60
**% anti-depressants**^‡^		45; 24.59%	3; 4.48%	18; 37.50%	24; 35.29%	**X**^**2**^ **= 18.15**	X^2^ = 0.0022	**2.04E–05/>53.00**	0.96/<1.60	**0.00051/9.51**	1.00/<1.60
% Anti-epileptics^‡^		3; 1.64%	0; 0.00%	2; 4.17%	1; 1.47%	X^2^ = 2.99E–31	X^2^ = 0.094	1.00/<1.60	0.76/<1.60	1.00/<1.60	1.00/<1.60
**% Anti-psychotics**^‡^		16; 8.74%	0; 0.0%	7; 14.58%	9; 13.23%	**X**^**2**^ **= 7.49**	X^2^ = 1.33E–31	**0.0062/16.82**	1.00/<1.60	0.16/<1.60	1.00/<1.60
**AQ–10 total score**^†^		3.26 ± 2.21	2.30 ± 1.75	3.52 ± 2.16	4.05 ± 2.32	**−4.05**	1.68	**9.19E–05/>53.00**	0.096/1.64	**0.0023/2.63**	1.00/<1.60
**HADS total score**^†^		14.18 ± 8.18	7.95 ± 5.66	16.23 ± 7.27	19.07 ± 6.89	**−7.83**	**−2.36**	**2.22E–12/>53.00**	**0.021/4.57**	**5.55E–11/>53.00**	0.52/<1.60
**OCI total score**^†^		15.62 ± 14.00	7.12 ± 7.98	21.29 ± 15.66	19.73 ± 13.56	**−5.26**	−0.18	**6.47E–07/>53.00**	0.86/<1.60	**1.62E–05/>53.00**	1.00/<1.60
**WSAS total score**^†^		12.17 ± 11.67	2.86 ± 6.44	15.18 ± 10.39	19.22 ± 10.48	**−8.87**	−1.95	**8.18E–15/>53.00**	0.054/2.33	**2.05E–13/>53.00**	1.00/<1.60
Central coherence total score^†^		1.31 ± 0.39	1.30 ± 0.40	1.33 ± 0.41	1.31 ± 0.38	−0.50	−1.28	0.62/<1.60	0.21/<1.60	1.00/<1.60	1.00/<1.60
**TAS–20 score**^†^		51.55 ± 15.64	35.94 ± 10.89	51.26 ± 14.10	58.95 ± 13.17	**−2.23**	0.061	**0.028/3.71**	0.95/<1.60	0.69/<1.60	1.00/<1.60
RSAS score^†^		12.91 ± 8.47	8.56 ± 6.95	13.00 ± 7.96	14.92 ± 8.85	−1.57	0.092	0.12/<1.60	0.93/<1.60	1.00/<1.60	1.00/<1.60

Notes: Results in bold indicate a significant difference between groups, either between HCs versus ac-AN (^a^), or WR versus ac-AN (^b^).

^1^ – Original data, reported in mean ± SD; ^2^ – Transformed data (log), reported in median ± range; ^a^ – HC versus ac-AN; ^b^ – WR versus ac-AN; † − Mann–Whitney U Test; ‡ − Two-Proportion Z Test (reported in X^2^).

Abbreviations: ac-AN, Acute Anorexia Nervosa; ADOS, Autism Diagnostic Observation Schedule; BF, Bayes Factor; Bon., Bonferroni; BMI, Body Mass Index; Comm., Communication; AQ, Autism Quotient; EDE-Q, Eating Disorder Examination; HADS, Hospital Anxiety and Depression Scale; HC, Healthy Control; IQ, Intelligence Quotient; OCI, Obsessive Compulsive Inventory; RSAS, Revised Social Anhedonia Scale; TAS, Toronto Alexithymia Scale; WR, Weight Restored; WSAS, Work and Social Adjustment Scale; Y, Years.

### Differences in structural parameters across diagnostic groups

**Figure 1.**
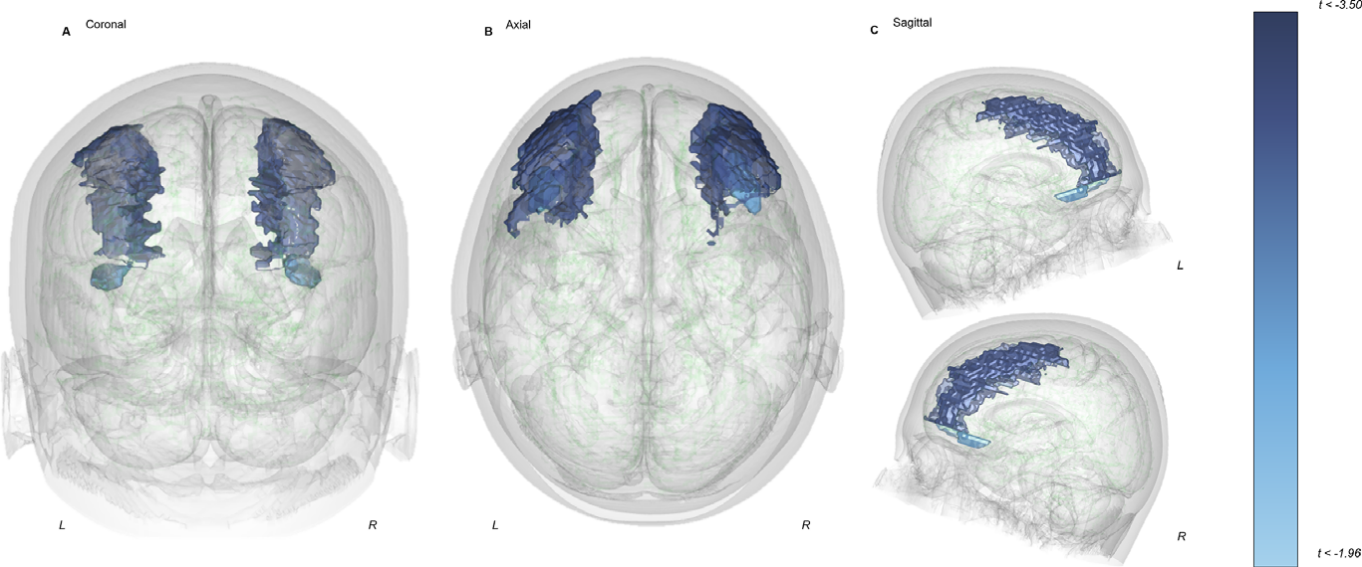
A priori differences in MFG and lateral OFC volume between those with ac-AN (n = 68) and HCs (n = 67) in coronal (a), axial (b), and sagittal (c) orientations. Findings delineate reduced volume in those with ac-AN relative to HCs. Abbreviations: L, Left; R, Right.

Between-group structural comparison identified significant differences within a priori areas. In those with ac-AN relative to HCs, the MFG region was smaller in volume (t = −3.833; p = 2.03E−04) and surface area (t = −3.304; p = 0.0013). The OFC also exhibited smaller volume (t = −2.281; p = 0.024) and surface area (t = −2.727; p = 0.00734) in ac-AN versus HC groups (Figure 1; Table 2). There were no further differences in a priori structural brain parameters when comparing ac-AN and HC groups. Whole brain analyses did not identify significant a priori or exploratory group-based differences in ac-AN versus WR groups post-Bonferroni correction (Supplementary Figure 1; Supplementary Table 2).

**Table 2.** Differences in a priori volume, surface area and cortical thickness in those with ac-AN (n = 68) versus HCs (n = 67), and those with ac-AN versus WR (n = 48)

Region (Volume)	HC (n = 67)	WR (n = 48)	ac-AN (n = 68)	t^a^	t^b^	p^a^/BF^a^	p^b^/BF^b^
MF caudal	0.0046 ± 6.06E–04	0.0048 ± 0.00061	0.0045 ± 0.00063	1.60	1.56	0.11/<1.60	0.12/<1.60
**MF rostral**	**0.012 ± 0.0013**^**1**^**;−4.43 ± 0.54**^**2**^	0.012 ± 0.0016^1^;−4.39 ± 0.57^2^	**0.012 ± 0.0014**^**1**^**;−4.46 ± 0.51**^**2**^	**3.83**	1.37	**2.03E–04/>53.00**	0.17/<1.60
ACC caudal	0.0014 ± 2.17E–04^1^;−6.56 ± 0.75^2^	0.0014 ± 1.82E–04^1^;−6.58 ± 0.58^2^	0.0014 ± 2.31E–04^1^;−6.57 ± 0.73^2^	1.82	0.90	0.072/1.95	0.37/<1.60
ACC rostral	0.0017 ± 2.43E–04	0.0018 ± 2.13E–04	0.0017 ± 2.36E–04	1.87	1.73	0.065/2.08	0.087/1.74
CC Mid anterior	3.75E–04 ± 7.63E–05^1^;−7.94 ± 0.90^2^	4.05E–04 ± 1.06E–04^^1^; −7.88 ± 1.01^2^	3.85E–04 ± 1.00E–04^1^;−7.91 ± 1.11^2^	−0.28	1.23	0.78/<1.60	0.22/<1.60
CC anterior	6.12E–04 ± 8.22E–05	6.05E–04 ± 7.22E–05	6.051E–04 ± 9.91E–05	0.53	0.30	0.60/<1.60	0.77/<1.60
**OFC lateral**	**0.0060 ± 5.89**	0.0061 ± 5.98E–04	**0.0059 ± 5.89E–04**	**2.28**	1.83	**0.024/4.07**	0.071/1.96
OFC medial	0.0040 ± 3.70E–04^1^;−5.53 ± 0.39^2^	0.0040 ± 3.89E–04^1^;−5.52 ± 0.39^2^	0.0040 ± 3.19E–04^1^;−5.53 ± 0.33^2^	1.63	1.77	0.11/<1.60	0.078/1.85

Region (Area)	HC (n = 67)	WR (n = 48)	ac-AN (n = 68)	t^a^	t^b^	p^a^/BF^a^	p^b^/BF^b^
MF caudal	0.0015 ± 1.76E–04^1^;−6.48 ± 0.11^2^	0.0016 ± 1.85E–04^1^;−6.44 ± 0.11^2^	0.0016 ± 1.84E–04^1^;−6.47 ± 0.12^2^	0.54	0.81	0.59/<1.60	0.42/<1.60
**MF rostral**	**0.0041 ± 3.62E–04**	0.0041 ± 4.31E–04	**0.0040 ± 3.89E–04**	**3.30**	0.76	**0.001343.76**	0.45/<1.60
ACC caudal	4.61E–04 ± 5.99E–05	4.52E–04 ± 5.01E–05	4.52E–04 ± 6.40E–05	1.50	−0.03	0.14/<1.60	0.98/<1.60
ACC rostral	4.84E–04 ± 6.44E–05	4.93E–04 ± 5.77E–05	4.80E–04 ± 6.34E–05	1.39	0.58	0.17/<1.60	0.57/<1.60
**OFC lateral**	**0.0020 ± 1.73E–04**	0.0020 ± 1.995E–04	**0.0019 ± 1.78E–04**	**2.73**	1.53	**0.0073/10.20**	0.13/<1.60
OFC Medial	0.0013 ± 1.00E–04^1^;−6.61 ± 0.073^2^	0.0013 ± 1.05E–04^1^;−6.61 ± 0.076^2^	0.0013 ± 9.27E–05^1^;−6.62 ± 0.070^2^	0.43	0.67	0.67/<1.60	0.51/<1.60

Region (Thickness)	HC (n = 67)	WR (n = 48)	ac-AN (n = 68)	t^a^	t^b^	p^a^/BF^a^	p^b^/BF^b^
MF caudal	2.55 ± 0.13	2.58 ± 0.16	2.54 ± 0.12	0.69	0.93	0.49/<1.60	0.36/<1.60
MF rostral	2.35 ± 0.12	2.39 ± 0.12	2.36 ± 0.098	0.28	1.21	0.78/<1.60	0.23/<1.60
ACC caudal†	2.76 ± 0.23	2.79 ± 0.20	2.74 ± 0.18	0.71	0.25	0.48/<1.60	0.080/1.822
ACC rostral	3.09 ± 0.19	3.02 ± 0.20	3.06 ± 0.17	0.98	1.15	0.33/<1.60	0.25/1.06
OFC lateral	2.78 ± 0.13	2.82 ± 0.15	2.79 ± 0.13	−0.14	0.91	0.89/<1.60	0.37/<1.60
OFC medial†	2.60 ± 0.12	2.63 ± 0.16	2.59 ± 0.12	1.36	1.79	0.18/<1.60	0.078/1.85

Notes: Regions (in mm^3^) in bold indicate a significant difference between groups, either between HCs versus ac-AN (^a^), or WR versus ac-AN (^b^). ^1^ – Original data, reported in mean ± SD; ^2^ – Transformed data (log), reported in median ± range; ^a^ – HC versus ac-AN; ^b^ – WR versus ac-AN; † − Mann–Whitney U Test.

Abbreviations: ac-AN, Acute Anorexia Nervosa; ACC, Anterior Cingulate Cortex; BF, Bayes Factor; CC, Cingulate Cortex; HC, Healthy Control; MF, Medial Frontal; OFC, Orbitofrontal Cortex; WR, Weight Restored.

### Associations between structural parameters and autistic characteristics

**Figure 2.**
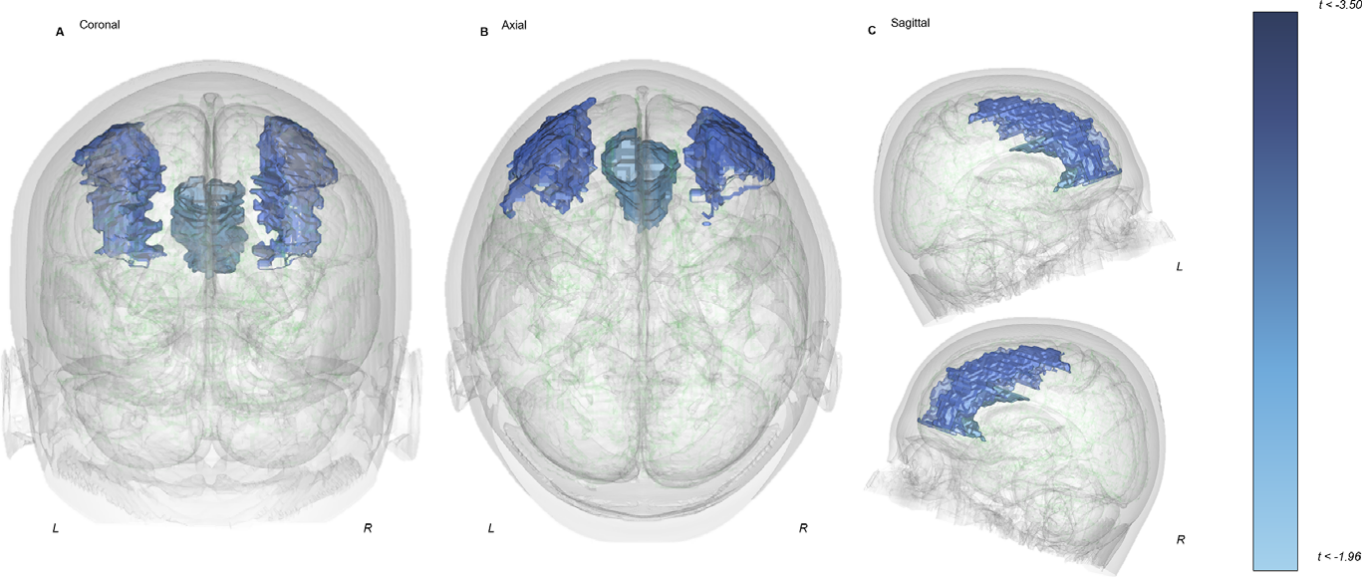
A priori associations between brain volume and AQ-10 scores in those with ac-AN (n = 68) and those who are WR (n = 48) in coronal (a), axial (b), and sagittal (c) orientations. Findings delineate a negative correlation within the rostral MFG and rostral ACC relative to increase in AQ-10 scores. Abbreviations: L, Left; R, Right.

Evaluation of a priori structural associations alongside AQ-10 scores identified regions of interest in the combined ac-AN and WR group (n = 116). Rostral MFG (β = −0.21; p = 0.016) and rostral ACC (β = −0.19; p = 0.038) volume were inversely associated with AQ-10 scores (Figure 2; Table 3). No additional structural parameters were associated with AQ-10 scores, and no exploratory associations survived post hoc correction in this group (Supplementary Figure 2; Supplementary Table 3). Analysis assumptions of linearity between the independent variable, mediator, and dependent variable (Baron & Kenny, 1986) could not be met to conduct mediation analyses via both a priori and agnostic approaches. Lastly, the relationship between rostral MFG surface area and AQ-10 scores significantly differed between the ac-AN and WR groups, with a significantly steeper negative slope seen in WR relative to ac-AN individuals (Supplementary Table 3). The exploratory analysis identified further significant differences in associations between structural parameters and AQ-10 scores within the cerebrospinal fluid and lateral occipital lobe thickness (Supplementary Table 4).

**Table 3.** Associations between brain structure and autistic characteristics via AQ-10 scores in those WR and in those with ac-AN (n = 116)

Region (Volume)	Estimate	CI	t	p/BF	R^2^
MF caudal	0.026	−0.16, 0.22	0.28	0.78/<1.60	0.11
**MF rostral**	**−0.21**	**−0.38, −0.041**	**−2.46**	**0.016/5.68**	**0.29**
ACC caudal	−0.029	−0.22, 0.17	−0.30	0.77/<1.60	0.060
**ACC rostral**	**−0.19**	**−0.37, −0.011**	**−2.10**	**0.038/2.95**	**0.18**
CC mid anterior	−0.064	−0.27, 0.14	−0.63	0.53/<1.60	−0.22
CC anterior	0.064	−0.14, 0.27	0.63	0.53/<1.60	−0.027
OFC lateral	−0.18	−0.37, 0.019	−1.96	0.052/2.38	0.16
OFC medial	−0.048	−0.24, 0.14	−0.51	0.61/<1.60	0.13

Region (Area)	Estimate	CI	t	p/BF	R^2^
MF caudal	−0.011	−0.21, 0.19	−0.11	0.91/<1.60	0.034
MF rostral	−0.17	−0.36, 0.019	−1.78	0.078/1.85	0.11
ACC caudal	−0.045	−0.25, 0.16	−0.44	0.66/<1.60	−0.012
ACC rostral	−0.17	−0.32, 0.071	−1.27	0.21/<1.60	0.032
OFC lateral	−0.16	−0.35, 0.037	−1.60	0.11/<1.60	0.056
OFC medial	0.086	−0.12, 0.29	0.84	0.40/<1.60	−0.025

Region (Thickness)	Estimate	CI	t	p/BF	R^2^
MF caudal	0.015	−0.18, 0.21	0.15	0.88/<1.60	0.088
MF rostral	−0.15	−0.33, 0.023	−1.72	0.088/1.72	0.23
ACC caudal	0.023	−0.18, 0.22	0.23	0.82/<1.60	−0.0047
ACC rostral	−0.11	−0.30, 0.075	−1.19	0.24/<1.60	0.12
OFC lateral	−0.076	−0.27, 0.12	−0.77	0.44/<1.60	0.056
OFC medial	−0.094	−0.28, 0.090	−1.01	0.31/<1.60	0.16

Notes: Regions (in mm^3^) in bold indicate a significant association between brain volume/surface area/cortical thickness and AQ-10 scores. All tests are non-parametric.

Abbreviations: ac-AN, Acute Anorexia Nervosa; ACC, Anterior Cingulate Cortex; BF, Bayes Factor; CC, Cingulate Cortex; CI, Confidence Interval; MF, Medial Frontal; OFC, Orbitofrontal Cortex; WR, Weight Restored].

### Volumetric structure and AQ-10 scores predicting future social behavior

A priori RFR models were less-than-ideal in their accuracy and ability to predict future levels of both TAS-20 and SAS scores, collected approximately 2.61 years after MRI scan acquisition. Both a higher R^2^ and percentage of variance explained indicate higher accuracy associated with model performance (Smith, Ganesh, & Liu, 2013), with respective values of 1.0 and 100% depicting perfect model performance. Out of 10 runs, the RFR model trained to predict TAS-20 scores was only able to explain an average of 46.7% of variance associated with score prediction, with an average OOB error of 11.2, MSE of 137.8 and R^2^ value of 0.37. The model used to predict SAS scores only explained an average of 38.0% of variance associated with score prediction, with an OOB error of 6.6 and an MSE value of 42.8 (Supplementary Table 5). The 10 most important variables associated with explaining MSE variation of TAS-20 score prediction consisted of SAS/TAS scores in TP1 (44.2%/42.2% respectively), time-of-scan BMI (6.7%), NART scores (6.2%), age (4.0%), rostral and caudal ACC surface area (3.4% and 1.2% respectively), rostral ACC volume (1.2%), BMI group classification (ac-AN/WR/HC; 0.6%), and caudal ACC volume (0.4%). Top 10 variables explaining the variation of SAS score prediction also consisted of SAS/TAS-20 scores in TP1 (70.7%/6.7% respectively), medial OFC thickness (3.9%), caudal ACC thickness (3.1%), rostral MFG volume (3.1%), lateral OFC thickness (2.7%), age (2.7%), medication (2.6%), rostral MFG surface area (2.2%), and ACC volume (0.1%) (Supplementary Figure 3). Importantly, the reported variables are associated with less-than-ideal model performance and may not serve as significant predictors of future social behavior via TAS-20 and SAS scores.

Exploratory RFR models slightly altered accuracy and ability to predict future TAS-20 and SAS scores but remained less-than-ideal. The TAS-20 model was able to explain an average of 43.9% of variance associated with score prediction, with an average OOB error of 11.4, an MSE of 127.6 and an R^2^ value of 0.33. The SAS model explained an average of 40.7% of variance associated with score prediction, as well as a lower OOB error of 6.4 and an MSE value of 42.4 (Supplementary Table 6). AQ-10 scores also fell outside of the 10 most important variables explaining variation in predicted response value (Supplementary Figure 4; Supplementary Materials [Results]).

## Discussion

This study utilized neuroimaging and questionnaire-based data from 183 individuals (nHC = 67; nWR = 48; nac-AN = 68) to investigate neuroanatomical associations in those with WR relative to those acutely underweight. Differences across diagnostic groups identified altered structure of the rostral MFG and lateral OFC across ac-AN and HC groups, but these differences were not significant when comparing ac-AN and WR groups. Associations between structural parameters and autistic characteristics in both the ac-AN and WR groups were identified within the rostral MFG and rostral ACC, with a significant difference in associations observed between the ac-AN and WR groups within the rostral MFG surface area. RFR models were less-than-ideal in their ability to predict future socioemotional behavior. Findings suggest that volumetric reduction of the rostral MFG is implicated in AN, and considering nonsignificant differences seen between ac-AN and WR individuals may not significantly recover upon weight restoration. Inverse correlations between ACC and MFG structure and the presence of autistic characteristics were conducted across those acutely ill and those with WR, suggesting these associations are present regardless of BMI state.

Findings firstly suggest that characteristics associated with autism are unrelated to weight restoration in AN. Differences between HCs and those with ac-AN consisted of autistic characteristics as described in both the AQ-10 and ADOS-2, reflecting previous work (Björnsdotter et al., 2018; Kerr-Gaffney et al., 2021; Westwood et al., 2016) and supporting a growing body of research highlighting a lack of clinical difference between AN and atypical AN (Golden & Walsh, 2024). Limited literature has explored the relationship between the presence of autistic characteristics and weight restoration in AN, but study findings corroborate recent reports suggesting that autistic characteristics remain stable across the AN condition and are unrelated to BMI (Boltri & Sapuppo, 2021; Nuyttens, Simons, Antrop, & Glazemakers, 2024).

Between-group findings identified that neuroanatomical differences between HCs and those with ac-AN did not persist when comparing WR with ac-AN individuals. Differences between ac-AN and HC groups were seen in the volume and surface area of the rostral MFG region and lateral OFC, which did not significantly differ between those acutely ill relative to WR individuals. The OFC is integral for the interpretation of taste and for the control of food-related intake, with its lateral subregion specifically integrating nonreward and punishment (Rolls, Cheng, & Feng, 2020). Studies examining functionality of the OFC suggest that this region is highly associated with altered and maladaptive decision-making during choice deliberation in AN (Xue et al., 2022). Orbitofrontal findings reflect reports from previous research, which identify altered structure of the left OFC in both ac-AN and WR individuals relative to HCs (Frank, Shott, Hagman, & Mittal, 2013; Sader, Williams, & Waiter, 2022). However, findings from this previous research report larger OFC volume relative to the findings of smaller volume seen in this research, which could be attributed to the sample-based differences across studies. From publications reporting larger brain volume in the previous meta-analysis, age and DOI were slightly higher (age: 20.96 ± 5.67; DOI: 5.65 ± 5.43) than what was seen in the current study (age: 18.75 ± 3.05; DOI: 3.37 ± 2.70) and BMI was slightly lower (15.73 ± 0.49 versus 16.60 ± 1.44) which may contribute towards the disparity in reports of OFC volume. Findings suggest that reports across studies indicate a differential structure of the OFC, that further research needs to investigate the directionality of structural differences alongside relationships with age, BMI and DOI. Reduced volume of the MFG in those with AN relative to HCs has been reported in previous research (Brodrick et al., 2021; Nickel et al., 2018; Sader, Williams, & Waiter, 2022) and do not demonstrate significant differences in those WR relative to those with ac-AN (Brodrick et al., 2021). The MFG is associated with levels of impulsivity (Pan et al., 2021) and inhibitory control (Gavazzi et al., 2021). Mechanisms contributing to inhibitory control are termed ‘proactive’, a top-down mechanism working to suppress pre-emptive actions, and ‘reactive’, a bottom-up mechanism of control, functioning as a trigger to cease already-initiated functions (Gavazzi et al., 2021). The MFG is reported as a region associated with both proactive and reactive means of inhibitory control (Gavazzi et al., 2021). Despite previous studies implicating differences in cortical thickness across a priori regions in AN (Brodrick et al., 2021; Lavagnino et al., 2018; Mishima et al., 2021; Nickel et al., 2018) and autism (Brodrick et al., 2021; Shen et al., 2024) research, findings suggest that cortical thickness in those with ac-AN relative to either HC or WR individuals is unrelated to the presentation of autistic characteristics.

Neuroanatomical associations with autistic characteristics in those with WR as well as in those with ac-AN identified rostral MFG and rostral ACC volume as demonstrating significant correlations alongside AQ-10 score, suggesting that regardless of BMI state, autistic characteristics are still associated with these structural parameters. The structural reduction of frontal regions is robustly reported in autistic relative to neurotypical individuals (Zielinski et al., 2014). The ACC, implicated in emotional regulation, response monitoring, and goal-directed behavior (Rolls, 2019), has also been reported as structurally reduced in Autistic relative to non-Autistic groups (Ecker et al., 2010; Laidi et al., 2019). Separate evaluation of ac-AN and WR groups identified significant differences in associations between rostral MFG surface area and autistic characteristics. Findings suggest that while restoration of BMI in ac-AN is not significantly associated with rostral MFG volume and autistic characteristics, the surface area of this region may play a different role. However, factors influencing brain morphology, such as hydration status or length of weight restoration, may also explain a difference in relationships between rostral MFG surface area and the presence of autistic characteristics, and further research would benefit from investigating these potential confounds.

While RFR models identified moderate-to-weak predictive performance, they lacked functional and diffusion-based data and may have performed adequately given the limited provided features. Additionally, autistic characteristics were unable to accurately predict future levels of alexithymia and social anhedonia, but a priori brain regions served as important predictive features. Despite strong associations between these features and autistic characteristics (Gadow & Garman, 2020; Kinnaird, Stewart, & Tchanturia, 2019; 2020), AQ-10 scores were not considered important predictors in a priori or exploratory RFR models. However, regions associated with the presentation of autistic characteristics, such as the ACC and MFG, were considered important predictive variables to predict scores in a priori models. The TAS-20 model identified surface area and volume of the caudal and rostral ACC, while the SAS model identified caudal ACC thickness, medial/lateral OFC thickness, rostral MFG surface area, and ACC volume to be important predictors. Identified regions have been reported in previous literature, in which levels of alexithymia were associated with ACC activity (van der Velde et al., 2013), and levels of social anhedonia were associated with middle prefrontal cortex and ACC activity (Healey et al., 2014), and OFC volume (Zhang et al., 2016).

### Limitations

There are limitations to consider for this study. Firstly, the WR group consisted of a range of individuals falling within different stages of AN recovery (i.e., fully recovered versus still displaying AN symptoms). As such, the WR group was termed as exhibiting weight restoration rather than full recovery from AN. Additionally, there was insufficient data to determine the duration of weight restoration in the WR group, and future research should incorporate these measures to assess whether a lack of differences in morphology is present in ac-AN and WR individuals irrespective of WR duration. The ac-AN group was recruited from multiple health services which displayed heterogeneity in clinical course, history, or level of ac-AN treatment (Halls et al., 2021), which may influence the presentation and distribution of socioemotional and cognitive traits, as well as the presence of autistic characteristics. Further work would benefit from adjusting or screening for clinical course and more detailed diagnostic history. As the sample was predominantly community-based, we were unable to strictly control the level of dietary intake prior to MRI scans, which has the potential to impact brain morphology measures.

Due to high levels of missing data on the ADOS-2 relative to the AQ-10, the AQ-10 was used to assess autistic traits in the current study despite poorer accuracy/specificity. The race of the study sample was 82.5% white, limiting the generalizability of study findings to a racially diverse population or those of a multi-racial origin. Lastly, there were no measurements to track either socioeconomic status as well as recovery from ac-AN across research time-points, and no opportunity to assess the longitudinal effects of weight restoration on social behavior. Importantly, BMI levels are unable to determine if weight restoration improves systemic or neural impairments incurred throughout the AN state concerning malnutrition, over-exercise, or stress, and future research would benefit exploring these behavioral nuances.

## Conclusion

This study contributes to a growing body of work aiming to disentangle the impact of autistic characteristics and weight restoration in those with AN. We identified significant differences in the presentation of autistic characteristics as reported by the AQ-10 in those with ac-AN relative to HC groups, which did not significantly differ in those with ac-AN versus WR individuals. We also identified a priori group-wise differences between those with ac-AN and HC individuals within the MFG and OFC, which were nonsignificant when comparing those with ac-AN relative to WR individuals. Autistic characteristics in those with AN were inversely associated with MFG and ACC volume, with significant differences in associations of the rostral MFG surface area seen across BMI groups. Exploratory and a priori RFR models did not depict ideal accuracy to predict future levels of social anhedonia and alexithymia, and AQ-10 scores were not considered important factors to predict future social anhedonia and alexithymia. Findings shed light on potential neuroanatomical underpinnings associated with both AN symptomatology and autistic characteristics, providing evidence that both behavioral measures of these characteristics and neuroanatomical associations are unrelated to changes in BMI.

## Supporting information

Sader et al. supplementary material Sader et al. supplementary material

## Data Availability

The data that support the findings of this study are available from the corresponding author upon reasonable request. Datasets used for previous BEACON research may be found within online repositories. The name of the repository and accession number may be found here: https://osf.io/tqysn/?view_only=69a0f2183df14f5e82f4b728fba50f34.

## Supplementary material

The supplementary material for this article can be found at http://doi.org/10.1017/S0033291725001047.
