# Supplementary material for: Neuroanatomical associations with autistic characteristics in those with acute anorexia nervosa and weight-restored individuals
Source: Psychol Med. 2025 Apr 28;55:e120. doi: 10.1017/S0033291725001047 (PMC12094641; doi:10.1017/S0033291725001047)
Supplement: Sader et al. supplementary material [file S0033291725001047sup001.docx]

**Neuroanatomical Associations with Autistic Characteristics in those with Acute Anorexia Nervosa and Weight-Restored Individuals**

**Supplementary Materials**

**2.0 Materials & Methods**

*2.1 Participant Characteristics*

Participants were screened for the presence of psychological or psychiatric comorbidity using the structured clinical interview for DSM-5-research version (SCID-5-RV; First, Williams, Karg & Spitzer, 2015). Participants were not excluded due to other diagnoses to retain the ecological validity of the study, which estimates the prevalence of psychiatric conditions at 16-25% in the UK (Bebbington & McManus, 2020).

*2.2 Clinical and Self-Report Measures*

The AQ-10 is a tool recommended for use in adults who are possibly Autistic with no presence of a learning disability and assists in determining whether an individual should undergo a more comprehensive autism assessment (Allison, Auyeung & Baron-Cohen, 2012). Scoring for the autism quotient (AQ-10) consists of 10 statements with four possible answer choices (‘Definitely agree’, ‘Slightly agree’, ‘Slightly disagree’, ‘Definitely disagree’). The AQ-10 scores 1 point for each question, in which 1 point is scored for the items “Definitely” or “Slightly agree” on items 1, 7 and 8, and 1 point is scored for the items “Definitely” or “Slightly disagree” on questions 2, 3, 4, 5, 6 and 9 containing a possible score range of 0-10. Participants completed the Autism Diagnostic Observation Schedule-2 (ADOS-2) with a researcher who was highly trained to administer Module 4 of the ADOS-2, corresponding to adolescent and adult examinees.

*2.3 Scanning Parameters and MRI Data*

The study was piloted to ensure that participants understood the purpose of the study. For further information on recruitment and scanning procedures, see Halls et al. (2021).

*2.4 Statistical Analysis*

Random forest regression (RFR) models were differentially constructed depending on a-priori versus agnostic assessment approaches. RFRs were also implemented separately for both a-priori and agnostic whole brain approaches. A-priori RFR models included age, national adult reading test (NART), duration-of-illness (DOI), Toronto Alexithymia Scale (TAS-20) and Social Anhedonia Scale (SAS) scores (research timepoint 1 [TP1]), either TAS-20 or SAS scores (from research timepoint 2 [TP2], depending on the model being tested) and surface area, thickness and volume features of a-priori regions (middle frontal gyrus [MFG]/anterior cingulate cortex [ACC]/orbitofrontal cortex [OFC]). TAS-20 and SAS at TP2 served as predicted values. Region agnostic analyses included surface area, thickness and volume of all brain regions and all behavioural features. Included brain volumes were uncorrected, but estimated total intracranial volume (eTIV) was included as a feature to ensure consideration of head size during model generation. RFR analysis was conducted using the “randomForest()” R package, with each RFR model assessment repeated and averaged across 10 trials. Data was partitioned according to a “training” set and a “test” set using the “caTools()” R package. Training and test sets consisted of an 80% to 20% split respectively with use of 1,000 trees. The RFR models were tuned to assess the ideal number of variables to randomly sample as candidate at each node split which aims to establish the lowest “out-of-bag” error value, known as the “m_try_” value under the randomForest() package. For exploratory RFR models, the ideal m_try_ values were 81 and 162 for the TAS-20 and SAS RFR models respectively. A-priori RFRs identified ideal m_try_ values of 15 and 30 for the TAS-20 and SAS RFR models respectively. Accuracy and performance of the RFR models were reported using mean squared error, mean absolute error and R^2^ values.

**3.0 Results**

| **Supplementary Table 1. Differences in exploratory volume, surface area and cortical thickness in those with AAN (n=68) versus HCs (n=67), and those with AAN versus WR (n=48)** | | | | | | | | | |
| --- | --- | --- | --- | --- | --- | --- | --- | --- | --- |
| **Region (Volume)** | **HC (n=67)** | **WR (n=48)** | **AAN (n=68)** | **t^a^** | **t^b^** | **p^a^/BF^a^** | **p^b^/BF^b^** | **p(Bon)^a^/BF^a^** | **p(Bon)^b^/BF^b^** |
| eTIV | 1370962.64±111973.89 | 1376128.75±123840.61 | 1364072.26±121987.68 | 0.463 | 0.291 | 0.644/<1.600 | 0.772/<1.600 | 1/<1.600 | 1/<1.600 |
| **GM Cortex** | **0.182±0.0117^1^; -1.715±0.312^2^** | 0.184±0.0142^1^; -1.694±0.3433^2^ | **0.178±0.0146^1^; -1.734±0.389^2^** | **2.96** | 1.82 | **0.00368/17.84** | 0.0723/1.94 | 0.239/<1.600 | 1/<1.600 |
| GM Subcort | 0.0413±0.00265^1^; -3.187±0.3111^2^ | 0.0413±0.00295^1^; -3.210±0.3009^2^ | 0.0405±0.00269^1^; -3.205±0.3134^2^ | 1.975 | 0.954 | 0.0506/2.44 | 0.343/<1.600 | 1/<1.600 | 1/<1.600 |
| **GM Total** | **0.4863±0.02825^1^; -0.7290±0.2803^2^** | 0.4924±0.03544^1^; -0.7187±0.3232^2^ | **0.4788±0.03567^1^; -0.7328±0.3690^2^** | **2.513** | 1.406 | **0.0133/6.40** | 0.163/<1.600 | 0.865/<1.600 | 1/<1.600 |
| WM Total | 0.156±0.0103^1^; -1.857±0.287^2^ | 0.155±0.0102^1^; -1.871±0.287^2^ | 0.153±0.0120^1^; -1.870±0.413^2^ | 1.713 | 0.95 | 0.0893/1.71 | 0.345/<1.600 | 1/<1.600 | 1/<1.600 |
| MF Caudal | 0.004572±6.059E-04 | 0.004755±0.0006127 | 0.004516±0.0006333 | 1.6 | 1.558 | 0.112/<1.600 | 0.123/<1.600 | 1/<1.600 | 1/<1.600 |
| **MF Rostral** | **0.01209±0.001252^1^; -4.428±0.5357^2^** | 0.01218±0.001582^1^; -4.394±0.5664^2^ | **0.01166±0.001366^1^; -4.458±0.5148^2^** | **3.833** | 1.369 | **2.03E-04/>53.00** | 0.175/<1.600 | **0.0132/6.44** | 1/<1.600 |
| BanksSTS | 0.001856±2.577E-04 | 0.001867±2.751E-04 | 0.001818±3.163E-04 | 1.158 | 0.25 | 0.249/<1.600 | 0.803/<1.600 | 1/<1.600 | 1/<1.600 |
| Entorhinal | 0.001490±2.451E-04^1^; -6.523±0.7415^2^ | 0.001551±2.840E-04^1^; -6.507±0.8433^2^ | 0.001541±2.660E-04^1^; -6.514±0.9192^2^ | -0.91 | -0.723 | 0.365/<1.600 | 0.472/<1.600 | 1/<1.600 | 1/<1.600 |
| Fusiform | 0.007292±6.983E-04 | 0.007323±7.419E-04 | 0.007146±8.640E-04 | 1.799 | -0.058 | 0.0746/1.90 | 0.954/<1.600 | 1/<1.600 | 1/<1.600 |
| **Inferior Parietal** | **0.01062±0.001248** | **0.01069±0.001306** | **0.01013±0.001260** | **2.818** | **2.006** | **0.00565/12.58** | **0.0481/2.52** | 0.367/<1.600 | 1/<1.600 |
| Inferior Temporal | 0.008653±8.876E-04^1^; -4.755±0.1048^2^ | 0.008979±0.0011247^1^; -4.720±0.1187^2^ | 0.008663±0.0010278^1^; -4.756±0.1172^2^ | 0.615 | 0.856 | 0.539/<1.600 | 0.394/<1.600 | 1/<1.600 | 1/<1.600 |
| Lateral Occipital | 0.008936±9.425E-04^1^; -4.707±0.4461^2^ | 0.009135±0.001038^1^; -4.705±0.4703^2^ | 0.008931±0.001099^1^; 4.730±0.5748^2^ | 0.487 | 1.325 | 0.627/<1.600 | 0.189/<1.600 | 1/<1.600 | 1/<1.600 |
| Lingual† | 0.005217±6.642E-04 | 0.005150±7.391E-04 | 0.005126±6.124 | 0.386 | -0.76 | 0.700/<1.600 | 0.449/<1.600 | 1/<1.600 | 1/<1.600 |
| **Middle Temporal** | 0.009433±9.08E-04^1^; -4.668±0.5001^2^ | 0.009684±0.001179^1^; -4.656±0.5023^2^ | 0.009288±0.001145^1^; -4.701±0.5169^2^ | 1.449 | **2.221** | 0.150/<1.600 | **0.029/3.58** | 1/<1.600 | 1/<1.600 |
| Parahippocampal | 0.001621±2.042E-04 | 0.001647±1.871E-04 | 0.001635±1.831E-04 | -0.23 | -0.96 | 0.819/<1.600 | 0.340/<1.600 | 1/<1.600 | 1/<1.600 |
| Paracentral† | 0.002737±2.787E-04 | 0.002706±2.910E-04 | 0.002688±3.537E-04 | 1.973 | 0.244 | 0.0507/2.43 | 0.808/<1.600 | 1/<1.600 | 1/<1.600 |
| **Precentral** | **0.01004±7.723E-04** | 0.01015±8.650E-04 | **0.009853±0.001063** | **2.168** | 0.666 | **0.0322/3.33** | 0.507/<1.600 | 1/<1.600 | 1/<1.600 |
| **Postcentral** | **0.007099±7.132E-04** | 0.007103±7.155E-04 | **0.006681±7.832E-04** | **2.376** | 0.712 | **0.0191/4.87** | 0.478/<1.600 | 1/<1.600 | 1/<1.600 |
| **Precuneus** | **0.007123±6.013E-04** | 0.007254±6.970E-04 | **0.006993±0.0007642** | **2.037** | 1.201 | **0.0439/2.68** | 0.233/<1.600 | 1/<1.600 | 1/<1.600 |
| Cuneus | 0.002340±3.050E-04 | 0.002446±3.813E-04 | 0.002339±2.277E-04 | -0.6 | 0.691 | 0.549/<1.600 | 0.492/<1.600 | 1/<1.600 | 1/<1.600 |
| Superior Frontal | 0.01658±0.001566^1^;  -4.109±0.4632^2^ | 0.01688±0.001849^1^;  -0.4095±0.5529^2^ | 0.01630±0.001708^1^;  -4.123±0.4680^2^ | 1.96 | 1.74 | 0.0523/2.38 | 0.0854/<1.600 | 1/<1.600 | 1/<1.600 |
| **Superior Parietal** | **0.009819±0.001070** | 0.009803±0.001122 | **0.009598±0.001117** | **2.127** | 0.301 | **0.0354/3.11** | 0.764/<1.600 | 1/<1.600 | 1/<1.600 |
| **Superior Temporal** | 0.009578±0.001015^1^;  -4.652±0.6366^2^ | **0.009706±0.001141^1^;**  **-4.650±0.4390^2^** | **0.009271±0.001059^1^;**  **-4.700±0.5518^2^** | 1.949 | **2.169** | 0.0536/2.35 | **0.0329/3.27** | 1/<1.600 | 1/<1.600 |
| Brainstem | 0.01387±0.001206 | 0.01378±9.490E-04 | 0.01361±0.001090 | 1.117 | 0.837 | 0.266/<1.600 | 0.405/<1.600 | 1/<1.600 | 1/<1.600 |
| CSF† | 7.473E-04±1.491E-04 | 7.504E-04±1.794E-04 | 7.450E-04±1.333E-04 | 0.204 | -0.341 | 0.839/<1.600 | 0.734/<1.600 | 1/<1.600 | 1/<1.600 |
| Ventral DC | 0.002816±2.184E-04 | 0.002815±1.948E-04 | 0.002790±2.11E-04 | 1.007 | 0.317 | 0.316/<1.600 | 0.752/<1.600 | 1/<1.600 | 1/<1.600 |
| Vessel† | 2.456E-05±1.879E-05 | 2.179E-05±1.285E-05 | 2.353E-05±1.558E-05 | 0.03 | 0.079 | 0.976/<1.600 | 0.937/<1.600 | 1/<1.600 | 1/<1.600 |
| Choroid Plexus | 3.044E-04±7.291E-05^1^;  -8.127±1.059^2^ | 3.079E-04±7.231E-05^1^;  -8.097±1.124^2^ | 3.260E-04±9.339E-05^1^;  -8.063±1.172^2^ | -0.946 | -1.355 | 0.346/<1.600 | 0.179/<1.600 | 1/<1.600 | 1/<1.600 |
| **Supratentorial**† | **0.7263±0.03504** | 0.7295±0.04507 | **0.7136±0.04342** | **2.893** | 1.612 | **0.00454/15.02** | 0.111/<1.600 | 0.295/<1.600 | 1/<1.600 |
| **Supratentorial (Not Ventricle)**‡ | **0.7179±0.03525^1^;**  **-0.006988±4.375^2^** | 0.7213±0.04462^1^; 0.1705±4.496^2^ | **0.7041±0.04479^1^;**  **-0.3096±5.658^2^** | **2.987** | 1.596 | **0.00342/18.94** | 0.114/<1.600 | 0.222/<1.600 | 1/<1.600 |
| **Parsopercularis** | 0.003352±4.499E-04^1^;  -5.706±0.6254^2^ | **0.003572±5.138E-04^1^;**  **-5.653±0.6684^2^** | **0.003311±3.975E-04^1^;**  **-5.724±0.5228^2^** | 1.287 | **2.935** | 0.200/<1.600 | **0.00428/15.76** | 1/<1.600 | 1/<1.600 |
| Parsorbitalis† | 0.002181±2.811E-04 | 0.002192±3.436E-04 | 0.002161±3.141E-04 | 1.41 | 0.353 | 0.161/<1.600 | 0.725/<1.600 | 1/<1.600 | 1/<1.600 |
| **Parstriangularis** | **0.003195±4.280E-04^1^;**  **-5.780±0.6134^2^** | 0.003220±5.082E-04^1^;  -5.738±0.5780^2^ | **0.003075±4.205E-04^1^;**  **-5.789±0.6308^2^** | **2.041** | 1.76 | **0.0434/4.07** | 0.082/1.79 | 1/<1.600 | 1/<1.600 |
| Pericalcarine | 0.001608±3.087E-04 | 0.001603±3.050E-04 | 0.001586±2.651E-04 | -0.372 | -0.09 | 0.710/<1.600 | 0.929/<1.600 | 1/<1.600 | 1/<1.600 |
| **Supramarginal** | **0.008436±9.977E-04** | 0.008344±0.00101 | **0.007981±0.001010** | **3.746** | 1.117 | **2.78E-04 />53.00** | 0.267/<1.600 | 1/<1.600 | 1/<1.600 |
| ACC Caudal | 0.001415±2.17E-04^1^;  -6.575±0.7465^2^ | 0.001414±1.824E-04^1^;  -6.581±0.5821^2^ | 0.001375±2.311E-04^1^;  -6.596±0.7337^2^ | 1.818 | 0.902 | 0.0716/1.95 | 0.370/<1.600 | 1/<1.600 | 1/<1.600 |
| ACC Rostral | 0.001714±2.43E-04 | 0.001780±2.134E-04 | 0.00169±2.360E-04 | 1.865 | 1.734 | 0.0647/2.08 | 0.0865/1.74 | 1/<1.600 | 1/<1.600 |
| Insula | 0.005205±4.528E-04^1^;  -5.272±0.3436^2^ | 0.005284±5.297E-04^1^;  -5.257±0.4598^2^ | 0.005136±4.385E-04^1^;  -5.270±0.3874^2^ | 1.343 | 1.362 | 0.182/<1.600 | 0.177/<1.600 | 1/<1.600 | 1/<1.600 |
| **OFC Lateral** | **0.006037±5.888** | 0.006149±5.979E-04 | **0.005914±5.898E-04** | **2.281** | 1.829 | **0.0243/4.073** | 0.0710/1.96 | 1/<1.600 | 1/<1.600 |
| OFC Medial | 0.004022±3.704E-04^1^;  -5.533±0.3935^2^ | 0.004054±3.885E-04^1^;  -5.515±0.3973^2^ | 0.003969±3.186E-04^1^;  -5.526±0.3344^2^ | 1.628 | 1.786 | 0.106/<1.600 | 0.0776/1.85 | 1/<1.600 | 1/<1.600 |
| Frontal Pole | 9.645E-04±1.486E-04^1^;  -6.963±0.5858^2^ | 9.805E-04±1.509E-04^1^;  -6.953±0.778^2^ | 9.482E-04±1.469E-04^1^;  -6.987±0.8243^2^ | 1.357 | 1.416 | 0.177/<1.600 | 0.160/<1.600 | 1/<1.600 | 1/<1.600 |
| Temporal Pole | 0.001874±2.570E-04 | 0.001870±2.401E-04 | 0.001861±2.878E-04 | 0.81 | 0.116 | 0.420/<1.600 | 0.908/<1.600 | 1/<1.600 | 1/<1.600 |
| Transverse Temporal | 8.023E-04±1.074E-04 | 8.242E-04±1.105E-04 | 7.816E-04±1.002E-04 | 1.458 | 1.924 | 0.148/<1.600 | 0.0577/2.24 | 1/<1.600 | 1/<1.600 |
| Isthmus Cingulate | 0.001930±2.174E-04^1^;  -6.266±0.4908^2^ | 0.001952±2.369E-04^1^;  -6.253±0.6678^2^ | 0.001878±2.363E-04^1^;  -6.277±0.5582^2^ | 1.023 | 1.049 | 0.308/<1.600 | 0.297/<1.600 | 1/<1.600 | 1/<1.600 |
| CC Posterior | 6.865E-04±9.144E-05^1^;  -7.297±0.5043^2^ | 6.675E-04±8.457E-05^1^;  -7.322±0.5005^2^ | 6.673E-04±1.079E-04^1^;  -7.335±0.5959^2^ | 1.519 | 0.854 | 0.131/<1.600 | 0.395/<1.600 | 1/<1.600 | 1/<1.600 |
| CC Mid Posterior | 3.761E-04±6.630E-05^1^;  -7.939±0.7778^2^ | 3.738E-04±7.280E-05^1^;  -7.902±0.9269^2^ | 3.808E-04±6.781E-05^1^;  -7.879±0.9638^2^ | -0.358 | 0.001 | 0.721/<1.600 | 0.999/<1.600 | 1/<1.600 | 1/<1.600 |
| CC Central | 4.344E-04±9.001E-05 | 4.407E-04±9.874E-05 | 4.331E-04±1.043E-04 | 0.231 | 0.589 | 0.818/<1.600 | 0.557/<1.600 | 1/<1.600 | 1/<1.600 |
| CC Mid Anterior | 3.747E-04±7.63E-05^1^;  -7.937±0.9041^2^ | 4.052E-04±1.059E-04^1^;  -7.878±1.0089^2^ | 3.854E-04±1.003E-04^1^;  -7.912±1.114^2^ | -0.281 | 1.231 | 0.779/<1.600 | 0.222/<1.600 | 1/<1.600 | 1/<1.600 |
| CC Anterior | 6.122E-04±8.223E-05 | 6.053E-04±7.220E-05 | 6.051E-04±9.909E-05 | 0.531 | 0.299 | 0.596/<1.600 | 0.765/<1.600 | 1/<1.600 | 1/<1.600 |
| Amygdala | 0.00126±1.29E-04^1^;  -6.686±0.4577^2^ | 0.00128±1.60E-04^1^;  -6.678±0.5293^2^ | 0.00124±1.15E-04^1^;  -6.690±0.4056^2^ | 0.568 | 1.189 | 0.571/<1.600 | 0.238/<1.600 | 1/<1.600 | 1/<1.600 |
| Hippocampus | 0.00304±2.85E-04 | 0.00303±2.36E-04 | 0.00298±2.52E-04 | 1.008 | 0.294 | 0.316/<1.600 | 0.769/<1.600 | 1/<1.600 | 1/<1.600 |
| CB GM | 0.0404±0.00365 | 0.0411±0.00401 | 0.0409±0.00385 | -0.376 | -0.475 | 0.707/<1.600 | 0.636/<1.600 | 1/<1.600 | 1/<1.600 |
| CB WM | 0.0100±0.00101 | 0.0100±0.00111 | 0.00977±0.000944 | 1.915 | 1.395 | 0.0578/<1.600 | 0.167/<1.600 | 1/<1.600 | 1/<1.600 |
| Caudate‡ | 0.00255±2.86E-04^1^; 0.09491±5.213^2^ | 0.00255±3.01E-04^1^;  -0.439±5.037^2^ | 0.00256±3.03E-04^1^;  -0.0383±5.291^2^ | 0.889 | -0.39 | 0.376/<1.600 | 0.698/<1.600 | 1/<1.600 | 1/<1.600 |
| **Putamen** | **0.00362±3.72E-04** | 0.00356±3.95E-04 | **0.00347±3.44E-04** | **2.036** | 0.879 | **0.0439/2.68** | 0.382/<1.600 | 1/<1.600 | 1/<1.600 |
| Pallidum | 0.00140±1.33E-04^1^;  -6.564±0.5223^2^ | 0.00140±1.52E-04^1^;  -6.600±0.4365^2^ | 0.00138±1.21E-04^1^;  -6.601±0.4220^2^ | 1.502 | 0.045 | 0.136/<1.600 | 0.964/<1.600 | 1/<1.600 | 1/<1.600 |
| Accumbens | 4.72E-04±5.45E-05 | 4.76E-04±6.11E-05 | 4.57E-04±6.97E-05 | 1.703 | 0.941 | 0.0911/<1.600 | 0.350/<1.600 | 1/<1.600 | 1/<1.600 |
| Thalamus Proper | 0.004939±3.824E-04^1^;  -5.314±0.3303^2^ | 0.004993±4.160E-04^1^;  -5.316±0.4059^2^ | 0.004865±4.296E-04^1^;  -5.338±0.3691^2^ | 1.274 | 1.56 | 0.205/<1.600 | 0.122/<1.600 | 1/<1.600 | 1/<1.600 |
| Lateral Ventricle | 0.00389±0.00147^1^;  -5.609±1.688^2^ | 0.00386±0.00194^1^;  -5.683±2.056^2^ | 0.00443±0.00199^1^;  -5.47±2.065^2^ | -1.243 | -1.741 | 0.216/<1.600 | 0.0852/<1.600 | 1/<1.600 | 1/<1.600 |
| **Inferior Lateral Ventricle**‡ | 0.0002055±6.850E-05^1^;  -0.06501±4.235^2^ | **0.0002085±8.601E-05^1^; -0.2239±5.131^2^** | **0.0002409±1.434E-04^1^; 0.1438±5.451^2^** | -1.352 | **-2.055** | 0.179/<1.600 | **0.0429/2.72** | 1/<1.600 | 1/<1.600 |
| **Third Ventricle** | **6.037E-04±1.48E-04^1^;**  **-7.452±1.217^2^** | **6.031E-04±1.11E-04^1^;**  **-7.415±0.9144^2^** | **6.870E-04±1.73E-04^1^;**  **-7.364±1.138^2^** | **-3.824** | **-2.132** | **2.10E-04 />53.00** | **0.0359/3.08** | **0.0137/6.26** | 1/<1.600 |
| Fourth Ventricle | 0.00114±3.036E-04^1^;  -6.841±1.143^2^ | 0.00112±2.924E-04^1^;  -6.849±1.222^2^ | 0.00113±2.506E-04^1^;  -6.825±1.071^2^ | -0.792 | -0.263 | 0.430/<1.600 | 0.794/<1.600 | 1/<1.600 | 1/<1.600 |
| Fifth Ventricle† | 7.45E-08±4.38E-07 | 3.85E-08±2.67E-07 | 1.15E-08±9.46 | 0.721 | 0.212 | 0.472/<1.600 | 0.832/<1.600 | 1/<1.600 | 1/<1.600 |
| **PCC** | **0.002416±2.440E-04^1^;**  **-6.039±0.5711^2^** | 0.002371±2.389E-04^1^;  -6.058±0.5007^2^ | **0.002383±2.772E-04^1^;**  **-6.045±0.6401^2^** | **2.491** | -0.742 | **0.0141/6.12** | 0.46034/<1.600 | 0.9165/<1.600 | 1/<1.600 |
| **Region (Area)** | **HC (n=67)** | **WR (n=48)** | **AAN (n=68)** | **t^a^** | **t^b^** | **p^a^/BF^a^** | **p^b^/BF^b^** | **p(Bon)^a^/BF^a^** | **p(Bon)^b^/BF^b^** |
| MF Caudal | 0.001540±1.763E-04^1^;  -6.482±0.1141^2^ | 0.001602±1.848E-04^1^;  -6.443±0.1123^2^ | 0.001561±1.842E-04^1^;  -6.470±0.1177^2^ | 0.541 | 0.814 | 0.589/<1.600 | 0.418/<1.600 | 1/<1.600 | 1/<1.600 |
| **MF Rostral** | **0.004139±3.619E-04** | 0.004098±4.306E-04 | **0.004005±3.893E-04** | **3.304** | 0.761 | **0.00126/43.76** | 0.449/<1.600 | **0.0441/2.67** | 1/<1.600 |
| Banks STS | 7.240E-04±9.075E-05 | 7.214E-04±9.199E-05 | 7.167E-04±9.926E-05 | 0.651 | -0.301 | 0.516/<1.600 | 0.764/<1.600 | 1/<1.600 | 1/<1.600 |
| Entorhinal | 3.084E-04±4.912E-05 | 3.288E-04±5.649E-05 | 3.210E-04±4.812E-05 | -1.273 | 0.022 | 0.205/<1.600 | 0.983/<1.600 | 1/<1.600 | 1/<1.600 |
| Fusiform | 0.002226±1.939E-04^1^;  -6.111±0.08759^2^ | 0.002232±2.090E-04^1^;  -6.109±0.09191^2^ | 0.002215±2.073E-04^1^;  -6.117±0.09061^2^ | 1.027 | -0.196 | 0.307/<1.600 | 0.845/<1.600 | 1/<1.600 | 1/<1.600 |
| Inferior Parietal | 0.003635±4.162E-04 | 0.003632±4.029E-04 | 0.003568±3.700E-04 | 1.119 | 0.539 | 0.265/<1.600 | 0.591/<1.600 | 1/<1.600 | 1/<1.600 |
| Inferior Temporal | 0.002460±2.420E-04^1^;  -6.012±0.09888^2^ | 0.002509±2.461E-04^1^;  -5.992±0.09312^2^ | 0.002471±2.571^1^;  -6.008±0.10192^2^ | 0.023 | 0.181 | 0.918/<1.600 | 0.856/<1.600 | 1/<1.600 | 1/<1.600 |
| Lateral Occipital | 0.003617±3.798E-04^1^;  -0.1371±4.724^2^ | 0.003694±4.077E-04^1^;  -0.09474±4.779^2^ | 0.003734±4.099E-04; 0.04400±5.183^2^ | -1.847 | 0.148 | 0.0673/2.02 | 0.882/<1.600 | 1/<1.600 | 1/<1.600 |
| Lingual† | 0.002182±2.535E-04 | 0.002115±2.826E-04 | 0.002166±2.074E-04 | 0.111 | -1.866 | 0.912/<1.600 | 0.0654/2.06 | 1/<1.600 | 1/<1.600 |
| Middle Temporal | 0.002491±2.374E-04^1^;  -6.010±0.4959^2^ | 0.002518±2.781E-04^1^;  -5.994±0.4687^2^ | 0.002464±2.464E-04^1^;  -6.024±0.4264^2^ | 0.646 | 1.558 | 0.520/<1.600 | 0.123/<1.600 | 1/<1.600 | 1/<1.600 |
| Parahippocampal‡ | 4.807E-04±5.386E-05^1^; 0.1598±1.0049^2^ | 4.689E-04±5.260E-05^1^;  -0.07914±1.0342^2^ | 4.669E-04±4.693E-05^1^;  -0.10163±0.9648^2^ | 1.488 | -0.144 | 0.139/<1.600 | 0.885/<1.600 | 1/<1.600 | 1/<1.600 |
| **Paracentral** | **0.001007±8.149E-05** | 9.702E-04±1.003E-04 | **9.897E-04±1.006E-04** | **2.258** | -1.616 | **0.0258/3.90** | 0.110/<1.600 | 0.902/<1.600 | 1/<1.600 |
| Precentral | 0.003377±2.387E-04^1^;  -5.693±0.07127^2^ | 0.003404±3.026E-04^1^;  -5.687±0.08858^2^ | 0.003362±3.108E-04^1^;  -5.699±0.09109^2^ | 1.274 | 0.175 | 0.205/<1.600 | 0.861/<1.600 | 1/<1.600 | 1/<1.600 |
| Postcentral | 0.002937±2.430E-04^1^;  -5.834±0.08217^2^ | 0.002912±2.624E-04^1^;  -5.843±0.08831^2^ | 0.002903±0.0002709^1^;  -5.846±0.09098^2^ | 1.423 | -0.336 | 0.157/<1.600 | 0.738/<1.600 | 1/<1.600 | 1/<1.600 |
| Precuneus | 0.002676±2.064E-04^1^;  -5.926±0.07636^2^ | 0.002668±2.390E-04^1^;  -5.930±0.08934^2^ | 0.002635±2.331E-04^1^;  -5.943±0.08712^2^ | 1.889 | 0.091 | 0.0613/2.15 | 0.928/<1.600 | 1/<1.600 | 1/<1.600 |
| Cuneus | 0.001045±1.117E-04^1^;  -6.869±0.1061^2^ | 0.001065±1.459E-04^1^;  -6.853±0.1309^2^ | 0.001058±1.243E-04^1^;  -6.859±0.1180^2^ | -1.301 | -0.255 | 0.196/<1.600 | 0.799/<1.600 | 1/<1.600 | 1/<1.600 |
| Superior Frontal | 0.005056±3.882E-04 | 0.005058±4.671E-04 | 0.005002±4.203E-04 | 1.469 | 0.742 | 0.145/<1.600 | 0.460/<1.600 | 1/<1.600 | 1/<1.600 |
| Superior Parietal‡ | 0.003869±3.428E-04^1^; 0.06383±0.9724^2^ | 0.003848±4.073E-04^1^;  -0.02403±1.0906^2^ | 0.003831±3.557E-04^1^;  -0.04593±0.9716^2^ | 1.577 | -0.586 | 0.118/<1.600 | 0.560/<1.600 | 1/<1.600 | 1/<1.600 |
| Superior Temporal† | 0.002790±2.508E-04 | 0.002816±3.159E-04 | 0.002735±2.377E-04 | 1.561 | 0.847 | 0.121/<1.600 | 0.399/<1.600 | 1/<1.600 | 1/<1.600 |
| Parsopercularis | 0.001077±1.314E-04^1^;  -6.858±0.5892^2^ | 0.001103±1.431^1^;  -6.829±0.5181^2^ | 0.001060±1.110E-04^1^;  -6.868±0.4814^2^ | 1.444 | 1.979 | 0.151/<1.600 | 0.051/2.42 | 1/<1.600 | 1/<1.600 |
| Parsorbitalis‡ | 5.5505±6.100E-05^1^; 0.05884±0.9464^2^ | 5.407E-04±6.580E-05^1^;  -0.13728±1.1248^2^ | 5.492E-05±5.939E-05^1^; 0.03893±0.9634^2^ | 1.527 | -0.97 | 0.129/<1.600 | 0.335/<1.600 | 1/<1.600 | 1/<1.600 |
| Parstriangularis | 0.001033±1.306E-04^1^;  -6.894±0.7460^2^ | 0.001028±1.404E-04^1^;  -6.891±0.5456^2^ | 0.001010±1.088E-04^1^;  -6.903±0.4971^2^ | 1.348 | 1.016 | 0.180/<1.600 | 0.312/<1.600 | 1/<1.600 | 1/<1.600 |
| Pericalcarine | 9.456E-05±1.618E-04^1^;  -6.978±0.1733^2^ | 9.411E-04±1.634E-04^1^;  -6.983±0.1690^2^ | 9.519E-04±1.289E-04^1^;  -6.966±0.1360^2^ | -1.079 | -0.557 | 0.283/<1.600 | 0.579/<1.600 | 1/<1.600 | 1/<1.600 |
| Supramarginal | 0.002856±3.483E-04^1^;  -5.865±0.1193^2^ | 0.002870±3.774E-04^1^;  -5.862±0.1292^2^ | 0.002807±3.717E-04^1^;  -5.885±0.1345^2^ | 1.621 | 0.322 | 0.108/<1.600 | 0.748/<1.600 | 1/<1.600 | 1/<1.600 |
| ACC Caudal | 4.605E-04±5.988E-05 | 4.518E-04±5.010E-05 | 4.519E-04±6.397E-05 | 1.5 | -0.03 | 0.136/<1.600 | 0.976/<1.600 | 1/<1.600 | 1/<1.600 |
| ACC Rostral | 4.839E-04±6.439E-05 | 4.932E-04±5.770E-05 | 4.801E-04±6.339E-05 | 1.388 | 0.577 | 0.168/<1.600 | 0.566/<1.600 | 1/<1.600 | 1/<1.600 |
| Insula | 0.001647±1.337E-04^1^;  -0.03993±4.291^2^ | 0.001632±1.661E-04^1^;  -0.1417±4.927^2^ | 0.001619±1.359E-04^1^;  -0.2326±3.882^2^ | 1.75 | 0.35 | 0.0827/<1.600 | 0.727/<1.600 | 1/<1.600 | 1/<1.600 |
| **OFC Lateral** | **0.001977±1.727E-04** | 0.001974±1.995E-04 | **0.001921±1.778** | **2.727** | 1.529 | **0.00734/10.20** | 0.130/<1.600 | 0.257/<1.600 | 1/<1.600 |
| OFC Medial | 0.001346±1.004E-04^1^;  -6.613±0.07303^2^ | 0.001348±1.045E-04^1^;  -6.612±0.07596^2^ | 0.001342±9.266E-05^1^;  -6.616±0.06941^2^ | 0.434 | 0.666 | 0.665/<1.600 | 0.507/<1.600 | 1/<1.600 | 1/<1.600 |
| **Transverse Temporal** | **2.840E-04±3.153E-05** | 2.825E-04±3.314E-05 | **2.729E-04±3.061E-05** | **2.191** | 0.897 | **0.0304/3.46** | 0.372/<1.600 | 1/<1.600 | 1/<1.600 |
| Isthmus Cingulate | 6.585E-04±6.375E-05 | 6.601E-04±8.018E-05 | 6.534E-04±7.585E-05 | -0.392 | 0.568 | 0.696/<1.600 | 0.571/<1.600 | 1/<1.600 | 1/<1.600 |
| Temporal Pole‡ | 3.288E-04±3.302E-05^1^;  -0.002204±0.9683^2^ | 3.297E-04±3.715E-051^1^; 0.019055±0.9572^2^ | 3.291E-04±3.800E-05^1^;  -0.015622±1.0722^2^ | 0.216 | -0.405 | 0.829/<1.600 | 0.687/<1.600 | 1/<1.600 | 1/<1.600 |
| Frontal Pole | 2.139E-04±1.732E-05^1^;  -8.449±0.4472^2^ | 2.170E-04±2.031E-05^1^;  -8.456±0.4355^2^ | 2.138E-04±2.004E-05^1^;  -8.461±0.4690^2^ | 0.967 | 0.603 | 0.335/<1.600 | 0.548/<1.600 | 1/<1.600 | 1/<1.600 |
| WM Surface Area† | 0.06123±0.003483 | 0.06133±0.004361 | 0.06067±0.003939 | 1.854 | 0.211 | 0.0662/2.05 | 0.834/<1.600 | 1/<1.600 | 1/<1.600 |
| PCC | 8.212E-04±6.913E-05 | 8.053E-04±7.888E-05 | 8.150E-04±8.299E-05 | 1.343 | -0.817 | 0.182/<1.600 | 0.416/<1.600 | 1/<1.600 | 1/<1.600 |
| **Region (Thickness)** | **HC (n=67)** | **WR (n=48)** | **AAN (n=68)** | **t^a^** | **t^b^** | **p^a^/BF^a^** | **p^b^/BF^b^** | **p(Bon)^a^/BF^a^** | **p(Bon)^b^/BF^b^** |
| MF Caudal | 2.549±0.1328 | 2.576±0.1586 | 2.543±0.1226 | 0.691 | 0.929 | 0.491/<1.600 | 0.355/<1.600 | 1/<1.600 | 1/<1.600 |
| MF Rostral | 2.349±0.1169 | 2.386±0.1225 | 2.355±0.09753 | 0.282 | 1.205 | 0.778/<1.600 | 0.232/<1.600 | 1/<1.600 | 1/<1.600 |
| **Banks STS** | **2.661±0.1416** | 2.688±0.1581 | **2.618±0.1602** | **2.223** | 1.735 | **0.0281/3.67** | 0.0863/1.74 | 0.984/<1.600 | 1/<1.600 |
| Entorhinal | 3.507±0.2792 | 3.455±0.2739 | 3.482±0.2932 | 1.239 | -1.059 | 0.218/<1.600 | 0.293/<1.600 | 1/<1.600 | 1/<1.600 |
| **Fusiform** | **2.888±0.1014** | 2.893±0.1098 | **2.842±0.1166** | **2.849** | 1.361 | **0.00517/13.52** | 0.177/<1.600 | 0.181/<1.600 | 1/<1.600 |
| **Inferior Parietal** | **2.477±0.1294** | **2.489±0.1161** | **2.426±0.1302** | **3.174** | **2.882** | **0.00191/30.76** | **0.00499/13.91** | 0.0669/2.033 | 0.747/<1.600 |
| **Inferior Temporal** | **2.946±0.1334^1^; 1.079±0.04558^2^** | **2.964±0.1622^1^; 1.085±0.05389^2^** | **2.897±0.1145^1^; 1.063±0.03956^2^** | **3.073** | **2.311** | **0.00262/23.62** | **0.0232/4.21** | 0.0918/1.68 | 0.813/<1.600 |
| **Lateral Occipital** | **2.202±0.1061** | **2.195±0.1280** | **2.139±0.1082** | **4.47** | **2.333** | **1.79E-05 />53.00** | **0.022/4.38** | **6.27E-04 />53.00** | 0.770/<1.600 |
| Lingual | 2.236±0.1089 | 2.262±0.1205 | 2.201±0.1257 | 1.738 | 1.841 | 0.0848/1.76 | 0.0691/1.99 | 1/<1.600 | 1/<1.600 |
| **Middle Temporal** | **3.029±0.1192** | 3.055±0.1576 | **2.984±0.1521** | **3.01** | **2.323** | **0.00319/20.06** | **0.0225/4.31** | 0.112/<1.600 | 0.789/<1.600 |
| Parahippocampal | 2.956±0.2340 | 3.053±0.2063 | 3.020±0.2289 | -1.283 | -0.526 | 0.202/<1.600 | 0.600/<1.600 | 1/<1.600 | 1/<1.600 |
| Paracentral | 2.484±0.1371 | 2.536±0.1428 | 2.481±0.1534 | 0.606 | 1.302 | 0.545/<1.600 | 0.196/<1.600 | 1/<1.600 | 1/<1.600 |
| Precentral | 2.627±0.09949 | 2.645±0.12767 | 2.616±0.09996 | 1.173 | 0.561 | 0.243/<1.600 | 0.576/<1.600 | 1/<1.600 | 1/<1.600 |
| **Postcentral** | **2.134±0.1125** | 2.148±0.1175 | **2.102±01213** | **2.16** | 1.426 | **0.0327/3.29** | 0.157/<1.600 | 1/<1.600 | 1/<1.600 |
| **Precuneus** | 2.452±0.1176 | **2.498±0.1272** | **2.446±0.1319** | 0.758 | **2.097** | 0.450/<1.600 | **0.0389/2.91** | 1/<1.600 | 1/<1.600 |
| Cuneus | 2.031±0.1303 | 2.065±0.1357 | 2.009±0.1207 | 1.192 | 1.59 | 0.236/<1.600 | 0.115/<1.600 | 1/<1.600 | 1/<1.600 |
| Superior Frontal | 2.717±0.1186 | 2.766±0.1542 | 2.737±0.1265 | -0.285 | 0.805 | 0.776/<1.600 | 0.423/<1.600 | 1/<1.600 | 1/<1.600 |
| Superior Parietal | 2.191±0.1134 | 2.205±0.1252 | 2.172±0.1157 | 1.393 | 1.367 | 0.166/<1.600 | 0.175/<1.600 | 1/<1.600 | 1/<1.600 |
| **Superior Temporal** | **2.962±0.1314^1^; 1.085±0.04450^2^** | 2.976±0.1693^1^; 1.089±0.05591^2^ | **2.916±0.1385^1^; 1.069±0.04747^2^** | **2.156** | 1.883 | **0.0331/3.26** | 0.0631/2.11 | 1/<1.600 | 1/<1.600 |
| **Parsopercularis** | 2.696±0.1277 | **2.781±0.1451** | **2.698±0.1111** | 0.361 | **2.691** | 0.719/<1.600 | **0.00856/9.03** | 1/<1.600 | 0.300/<1.600 |
| **Parsorbitalis** | 2.874±0.1781 | **2.934±0.1793** | **2.842±0.1689** | 1.385 | **3.14** | 0.169/<1.600 | **0.00232/26.14** | 1/<1.600 | 0.0812/ |
| **Parstriangularis** | **2.589±0.1264** | **2.617±0.1293** | **2.557±0.1160** | **1.98** | **2.465** | **0.05/2.46** | **0.0157/5.64** | 1/<1.600 | 0.550/<1.600 |
| Pericalcarine | 1.815±0.1090 | 1.830±0.1090 | 1.780±0.1071 | 0.945 | 1.392 | 0.346/<1.600 | 0.167/<1.600 | 1/<1.600 | 1/<1.600 |
| **Supramarginal** | **2.583±0.1441** | 2.577±0.1484 | **2.523±0.1456** | **2.989** | 1.765 | **0.0034/19.04** | 0.0811/<1.600 | 0.119/<1.600 | 1/<1.600 |
| ACC Caudal† | 2.763±0.2270 | 2.790±0.2028 | 2.744±0.1757 | 0.711 | 0.252 | 0.479/<1.600 | 0.0802/1.82 | 1/<1.600 | 1/<1.600 |
| ACC Rostral | 3.087±0.1877 | 3.019±0.1971 | 3.059±0.1695 | 0.977 | 1.152 | 0.330/<1.600 | 0.253/<1.600 | 1/<1.600 | 1/<1.600 |
| **Insula** | 3.165±0.1251 | **3.213±0.1206** | **3.154±0.1217** | 0.992 | **2.147** | 0.323/<1.600 | **0.0346/3.16** | 1/<1.600 | 1/<1.600 |
| OFC Lateral | 2.784±0.1331 | 2.821±0.1454 | 2.788±0.1245 | -0.135 | 0.905 | 0.893/<1.600 | 0.368/<1.600 | 1/<1.600 | 1/<1.600 |
| OFC Medial† | 2.597±0.1209 | 2.625±0.1548 | 2.585±0.1203 | 1.36 | 1.786 | 0.176/<1.600 | 0.0776/1.85 | 1/<1.600 | 1/<1.600 |
| Transverse Temporal | 2.606±0.2005 | 0.2661±0.2096 | 2.622±0.1793 | 0.062 | 1.259 | 0.951/<1.600 | 0.212/<1.600 | 1/<1.600 | 1/<1.600 |
| **Isthmus Cingulate** | **2.614±0.1750** | 2.594±0.1372 | **2.562±0.1963** | **2.831** | 0.316 | **0.00545/12.95** | **0.753/<1.600** | 0.191/<1.600 | 1/<1.600 |
| Temporal Pole | 3.718±0.2679 | 3.716±0.2714 | 3.714±0.2718 | 0.595 | 0.472 | 0.553/<1.600 | 0.638/<1.600 | 1/<1.600 | 1/<1.600 |
| Frontal Pole | 2.862±0.1913^1^; 1.044±0.3495^2^ | 2.875±0.2214^1^; 1.044±0.3853^2^ | 2.858±02493^1^; 1.046±0.4816^2^ | 0.276 | 0.949 | 0.783/<1.600 | 0.345/<1.600 | 1/<1.600 | 1/<1.600 |
| **Mean Thickness** | **2.570±0.08851** | 2.594±0.10372 | **2.544±0.08765** | **2.767** | **2.503** | **0.00656/11.16** | **0.0142/6.09** | 0.230/<1.600 | 0.500/<1.600 |
| **PCC** | **2.648±0.1394** | 2.636±0.1334 | **2.618±0.1306** | **2.358** | 0.098 | **0.0200/4.70** | 0.922/<1.600 | 0.700/<1.600 | 1/<1.600 |

Footnotes: Regions (in mm^3^) in bold indicate a significant difference between cohorts, either between HCs vs. AAN (^a^), or WR vs. AAN (^b^). ^1^ – Original data, reported in mean ± SD; ^2^ – Transformed data (log), reported in median ± range; ^a^ – HC vs. AAN ; ^b^ – WR vs. AAN; † – Mann-Whitney U Test; ‡ – Standardised Box Cox Transformation

[Abbreviations: AAN – Acute Anorexia Nervosa; ACC – Anterior Cingulate Cortex; BF – Bayes Factor; CB – Cerebellum; CC – Cingulate Cortex; CSF – Cerebrospinal Fluid; eTIV – estimated Total Intracranial Volume; GM – Grey Matter; HC – Healthy Control; MF – Medial Frontal; OFC – Orbitofrontal Cortex; PCC – Posterior Cingulate Cortex; STS – Superior Temporal Sulcus; Subcort – Subcortical; WM – White Matter; WR – Weight Restored]


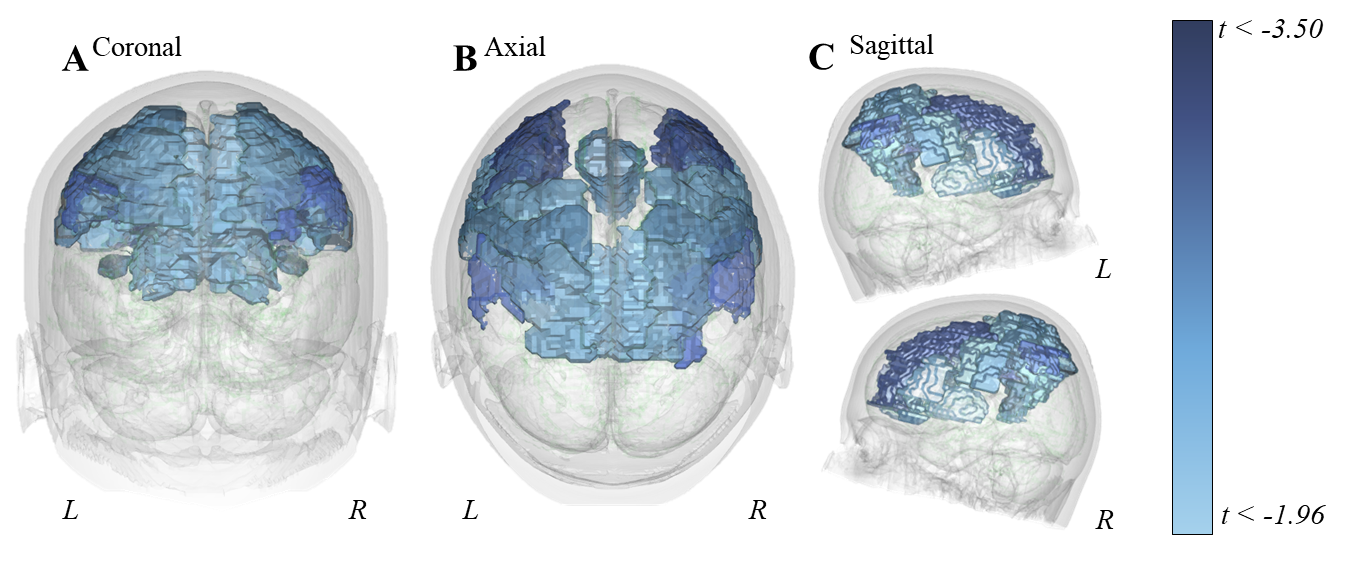


**Supplementary Figure 1.** Exploratory uncorrected differences in volume between those with AAN (n=68) and HCs (n=67) in coronal (A), axial (B) and sagittal (C) orientations. Findings delineate reduced volume in those with AAN relative to HCs [Abbreviations: L – Left; R – Right].

| **Supplementary Table 2. Associations between brain structure and autistic characteristics via AQ-10 scores in those WR and in those with AAN (n=116)** | | | | | | |
| --- | --- | --- | --- | --- | --- | --- |
| **Region (Volume)** | **Estimate** | **CI** | **t** | **p/BF** | **R^2^** | **p(Bon)/BF** |
| eTIV | 0.03847 | -0.1586, 0.2335 | 0.387 | 0.700/<1.600 | 0.03536 | 1/<1.600 |
| GM Cortex | -0.99266 | -0.2715, 0.07262 | -1.144 | 0.255/<1.600 | 0.2662 | 1/<1.600 |
| GM Subcort | -0.05747 | -0.2506, 0.1357 | -0.59 | 0.557/<1.600 | 0.07349 | 1/<1.600 |
| GM Total | -0.090035 | -0.2631, 0.08301 | -1.031 | 0.305/<1.600 | 0.2563 | 1/<1.600 |
| WM Total | 0.03884 | -0.1624, 0.2400 | 0.383 | 0.703/<1.600 | -0.00533 | 1/<1.600 |
| MF Caudal | 0.02635 | -0.1626, 0.2153 | 0.276 | 0.783/<1.600 | 0.1132 | 1/<1.600 |
| **MF Rostral** | **-0.20968** | **-0.3788, -0.04057** | **-2.457** | **0.0156/5.68** | **0.2898** | 1/<1.600 |
| BanksSTS | 0.08459 | -0.151, 0.2743 | 0.884 | 0.379/<1.600 | 0.106 | 1/<1.600 |
| Entorhinal | -0.16437 | -0.3552, 0.02643 | -1.707 | 0.0906/1.69 | 0.09588 | 1/<1.600 |
| Fusiform | 0.0001282 | -0.01951, 0.1953 | 0.001 | 0.999/<1.600 | 0.05384 | 1/<1.600 |
| Inferior Parietal | -0.05679 | -0.2496, 0.1360 | -0.584 | 0.561/<1.600 | 0.07707 | 1/<1.600 |
| Inferior Temporal | -0.05246 | -0.24386, 0.1389 | -0.543 | 0.588/<1.600 | 0.09029 | 1/<1.600 |
| Lateral Occipital | -0.01059 | -0.20636, 0.1852 | -0.107 | 0.915/<1.600 | 0.04823 | 1/<1.600 |
| Lingual | -0.07844 | -0.2809, 0.1240 | -0.768 | 0.444/<1.600 | -0.01748 | 1/<1.600 |
| Middle Temporal | -0.01323 | -0.1985, 0.1721 | -0.141 | 0.888/<1.600 | 0.1474 | 1/<1.600 |
| Parahippocampal | -0.10739 | -0.29088, 0.07611 | -1.16 | 0.249/<1.600 | 0.1638 | 1/<1.600 |
| Paracentral | -0.009702 | -0.1950, 0.1756 | -0.104 | 0.918/<1.600 | 0.1477 | 1/<1.600 |
| Precentral | -0.08712 | -0.2763, 0.1020 | -0.913 | 0.363/<1.600 | 0.1115 | 1/<1.600 |
| Postcentral | -0.001944 | -0.1941, 0.1902 | -0.02 | 0.984/<1.600 | 0.08327 | 1/<1.600 |
| Precuneus | -0.06298 | -0.2482, 0.1222 | -0.674 | 0.502/<1.600 | 0.1479 | 1/<1.600 |
| Cuneus | -0.1937 | -0.39178, .004297 | -1.939 | 0.0551/2.30 | 0.02603 | 1/<1.600 |
| Superior Frontal | -0.03287 | -0.2076, 0.01419 | -0.373 | 0.710/<1.600 | 0.2417 | 1/<1.600 |
| Superior Parietal | -0.08736 | -0.27733, 0.10261 | -0.911 | 0.364/<1.600 | 0.1038 | 1/<1.600 |
| Superior Temporal | -0.0981 | -0.2794, 0.08317 | -1.073 | 0.286/<1.600 | 0.184 | 1/<1.600 |
| Brainstem | -0.01436 | -0.2152, 0.1864 | -0.142 | 0.888/<1.600 | -0.00133 | 1/<1.600 |
| **CSF** | **-0.21199** | **-0.41182, -0.01216** | **-2.102** | **0.0378/2.97** | **0.008325** | 1/<1.600 |
| Ventral DC | 0.003517 | -0.1978, 0.2047 | 0.035 | 0.972/<1.600 | -0.00611 | 1/<1.600 |
| Vessel | 0.01363 | -0.1866, 0.2139 | 0.135 | 0.893/<1.600 | 4024 | 1/<1.600 |
| Choroid Plexus | -0.07514 | -0.2754, 0.1251 | -0.744 | 0.459/<1.600 | 0.00416 | 1/<1.600 |
| Supratentorial | -0.0423 | -0.2358, 0.1512 | -0.433 | 0.666/<1.600 | 0.06974 | 1/<1.600 |
| Supratentorial No Vent | -0.04189 | -0.2351 0.1513 | -0.43 | 0.668/<1.600 | 0.07285 | 1/<1.600 |
| Parsopercularis | -0.13869 | -0.3340, 0.05664 | -1.407 | 0.162/<1.600 | 0.05234 | 1/<1.600 |
| Parsorbitalis | -0.07944 | -0.2659, 0.1070 | -0.84 | 0.400/<1.600 | 0.1367 | 1/<1.600 |
| Parstriangularis | -0.1454 | -0.3336, 0.04291 | -1.53 | 0.129/<1.600 | 0.1197 | 1/<1.600 |
| Pericalcarine | -0.1494 | -0.3489, 0.05005 | -1.484 | 0.141/<1.600 | 0.01189 | 1/<1.600 |
| Supramarginal | -0.08408 | -0.2731, 0.1049 | -0.882 | 0.38/<1.600 | 0.113 | 1/<1.600 |
| ACC Caudal | -0.02915 | -0.2237, 0.1654 | -0.297 | 0.767/<1.600 | 0.05965 | 1/<1.600 |
| **ACC Rostral** | **-0.1926** | **-0.3745, -0.01072** | **-2.098** | **0.0382/2.95** | **0.178** | 1/<1.600 |
| **Insula** | **-0.26508** | **-0.453, -0.07714** | **-2.795** | **0.00612/11.80** | **0.1228** | 0.398/<1.600 |
| OFC Lateral | -0.182078 | -0.3661, 0.01947 | -1.961 | 0.0524/2.38 | 0.159 | 1/<1.600 |
| OFC Medial | -0.04845 | -0.2360, 0.1391 | -0.512 | 0.610/<1.600 | 0.1263 | 1/<1.600 |
| Frontal Pole | -0.1131 | -0.3016, 0.07536 | -1.189 | 0.237/<1.600 | 0.1177 | 1/<1.600 |
| Temporal Pole | -0.07771 | -0.2814, 0.1260 | -0.756 | 0.451/<1.600 | -0.03068 | 1/<1.600 |
| Transverse Temporal | -0.1487 | -0.34704, 0.049638 | -1.486 | 0.140/<1.600 | 0.02303 | 1/<1.600 |
| Isthmus Cingulate | -0.17334 | -0.3699, 0.02325 | -1.747 | 0.0833/<1.600 | 0.04023 | 1/<1.600 |
| CC Posterior | -0.04508 | -0.24405, 0.1539 | -0.449 | 0.654/<1.600 | 0.01681 | 1/<1.600 |
| CC Mid Posterior | -0.03245 | -0.2348, 0.1698 | -0.318 | 0.751/<1.600 | -0.0164 | 1/<1.600 |
| CC Central | 0.03121 | -0.1720, 0.2344 | 0.304 | 0.761/<1.600 | -0.02528 | 1/<1.600 |
| CC Mid Anterior | -0.06406 | -0.26689, 0.13876 | -0.626 | 0.533/<1.600 | -0.2162 | 1/<1.600 |
| CC Anterior | 0.06424 | -0.1392, 0.2676 | 0.626 | 0.533/<1.600 | -0.02739 | 1/<1.600 |
| Amygdala | 0.01825 | -0.1773, 0.2138 | 0.185 | 0.854/<1.600 | 0.0535 | 1/<1.600 |
| Hippocampus | -0.00269 | -0.2131, 0.1677 | -0.236 | 0.814/<1.600 | 0.09971 | 1/<1.600 |
| CB GM | 0.03056 | -0.1612, 0.2224 | 0.316 | 0.753/<1.600 | 0.08634 | 1/<1.600 |
| CB WM | 0.05502 | -0.1460, 0.2560 | 0.542 | 0.589/<1.600 | -0.00344 | 1/<1.600 |
| Caudate | -0.04321 | -0.2412, 0.15478 | -0.432 | 0.666/<1.600 | 0.02654 | 1/<1.600 |
| Putamen | -0.16024 | -0.3546, 0.03415 | -1.634 | 0.105/<1.600 | 0.06158 | 1/<1.600 |
| Pallidum | -0.04398 | -0.2468, 0.1588 | -0.43 | 0.668/<1.600 | -0.02127 | 1/<1.600 |
| Accumbens | 0.0585 | -0.14372, 0.2607 | 0.573 | 0.568/<1.600 | -0.01556 | 1/<1.600 |
| Thalamus Proper | -0.05836 | -0.25177, 0.1350 | -0.598 | 0.551/<1.600 | 0.07101 | 1/<1.600 |
| Lateral Ventricle | -0.0319 | -0.2342, 0.17038 | -0.312 | 0.755/<1.600 | -0.01611 | 1/<1.600 |
| Inferior Lateral Ventricle | -0.099491 | -0.3011, 0.1021 | -0.978 | 0.330/<1.600 | -0.00947 | 1/<1.600 |
| Third Ventricle | -0.142651 | -0.3459, 0.06063 | -1.391 | 0.167/<1.600 | -0.02623 | 1/<1.600 |
| Fourth Ventricle | 0.009726 | -0.1820, 0.2015 | 0.101 | 0.920/<1.600 | 0.0868 | 1/<1.600 |
| Fifth Ventricle | 0.03816 | -0.007494, 0.08381 | 1.656 | 0.100/<1.600 | -0.01796 | 1/<1.600 |
| PCC | -0.15231 | -0.3408, 0.0362 | -1.601 | 0.112/<1.600 | 0.1174 | 1/<1.600 |
| **Region (Area)** | **Estimate** | **CI** | **t** | **p** | **R^2^** | **p(Bon)** |
| MF Caudal | -0.01108 | -0.2083, 0.1861 | -0.111 | 0.912/<1.600 | 0.03391 | 1/<1.600 |
| MF Rostral | -0.1696 | -0.3587, 0.01941 | -1.778 | 0.0781/1.85 | 0.1125 | 1/<1.600 |
| Banks STS | 0.01075 | -0.08537, 0.30036 | 1.105 | 0.272/<1.600 | 0.07622 | 1/<1.600 |
| **Entorhinal** | **-0.21135** | **-0.4041, -0.01859** | **-2.173** | **0.0319/3.35** | **0.07731** | 1/<1.600 |
| Fusiform | 0.05566 | -0.1424, 0.2537 | 0.557 | 0.579/<1.600 | 0.02581 | 1/<1.600 |
| Inferior Parietal | -0.05051 | -0.2504, 0.1494 | -0.501 | 0.618/<1.600 | 0.007904 | 1/<1.600 |
| Inferior Temporal | -0.07288 | -0.2702, 0.1245 | -0.732 | 0.466/<1.600 | 0.03273 | 1/<1.600 |
| Lateral Occipital | -4.17E-02 | -0.2457, 0.1622 | -0.405 | 0.686/<1.600 | -0.033 | 1/<1.600 |
| Lingual | 0.01376 | -0.1905, 0.2180 | 0.133 | 0.894/<1.600 | -0.03637 | 1/<1.600 |
| Middle Temporal | -0.05352 | -0.2507, 0.1437 | -0.538 | 0.592/<1.600 | 0.03408 | 1/<1.600 |
| Parahippocampal | 0.04133 | -0.1501, 0.2327 | 0.428 | 0.670/<1.600 | 0.8991 | 1/<1.600 |
| Paracentral | -0.2817 | -0.2218, 0.1654 | -0.288 | 0.774/<1.600 | 0.0688 | 1/<1.600 |
| Precentral | -0.10862 | -0.3011, 0.08391 | -1.118 | 0.266/<1.600 | 0.07943 | 1/<1.600 |
| Postcentral | -0.01083 | -0.2076, 0.1859 | -0.109 | 0.913/<1.600 | 0.0386 | 1/<1.600 |
| Precuneus | -0.04104 | -0.2387, 0.1567 | -0.411 | 0.682/<1.600 | 0.02905 | 1/<1.600 |
| Cuneus | -0.09223 | -0.2940, 0.10958 | -0.906 | 0.367/<1.600 | -0.0114 | 1/<1.600 |
| Superior Frontal | -0.0388 | -0.2332, 0.1556 | -0.396 | 0.693/<1.600 | 0.06116 | 1/<1.600 |
| Superior Parietal | -0.08033 | -0.2717, 0.1110 | -0.832 | 0.407/<1.600 | 0.9071 | 1/<1.600 |
| Superior Temporal | -0.14331 | -0.3296, 0.04301 | -1.524 | 0.130/<1.600 | 0.1379 | 1/<1.600 |
| Parsopercularis | -0.08466 | -0.2867, 0.1174 | -0.83 | 0.408/<1.600 | -0.01362 | 1/<1.600 |
| Parsorbitalis | -0.5555 | -0.2496, 0.1386 | -0.567 | 0.572/<1.600 | 0.06396 | 1/<1.600 |
| Parstriangularis | -0.14138 | -0.3407, 0.05789 | -1.406 | 0.163/<1.600 | 0.01383 | 1/<1.600 |
| Pericalcarine | -0.04984 | -0.2521, 0.1525 | -0.488 | 0.616/<1.600 | -0.0164 | 1/<1.600 |
| Supramarginal | -0.07621 | -0.2697, 0.1173 | -0.78 | 0.437/<1.600 | 0.06995 | 1/<1.600 |
| ACC Caudal | -0.04506 | -0.2469, 0.15678 | -0.442 | 0.659/<1.600 | -0.01164 | 1/<1.600 |
| ACC Rostral | -0.12626 | -0.32366, 0.071138 | -1.268 | 0.208/<1.600 | 0.0323 | 1/<1.600 |
| Insula | -0.10613 | -0.30544, 0.093188 | -1.055 | 0.294/<1.600 | 0.01343 | 1/<1.600 |
| OFC Lateral | -0.15756 | -0.3525, 0.03739 | -1.602 | 0.112/<1.600 | 0.05615 | 1/<1.600 |
| OFC Medial | 0.08604 | -0.1171, 0.2892 | 0.839 | 0.403/<1.600 | -0.02516 | 1/<1.600 |
| Transverse Temporal | -0.13049 | -0.3257, 0.06468 | -1.325 | 0.188/<1.600 | 0.05394 | 1/<1.600 |
| Isthmus Cingulate | -0.1715 | -0.3729, 0.02996 | -1.687 | 0.0944/<1.600 | -0.0773 | 1/<1.600 |
| Temporal Pole | -0.17222 | -0.3695, 0.02504 | -1.73 | 0.0864/<1.600 | 0.03361 | 1/<1.600 |
| Frontal Pole | -0.07428 | -0.2665, 0.1179 | -0.766 | 0.445/<1.600 | 0.0825 | 1/<1.600 |
| WM Surface Area | -0.11392 | -0.3036, 0.07577 | -1.19 | 0.237/<1.600 | 0.1064 | 1/<1.600 |
| PCC | -0.08275 | -0.2818, 0.1163 | -0.824 | 0.412/<1.600 | 0.01586 | 1/<1.600 |
| **Region (Thickness)** | **Estimate** | **CI** | **t** | **p** | **R^2^** | **p(Bon)** |
| MF Caudal | 0.0148 | -0.1768, 0.2064 | 0.153 | 0.879/<1.600 | 0.08803 | 1/<1.600 |
| MF Rostral | -0.15301 | -0.3292, 0.02322 | -1.721 | 0.0881/1.72 | 0.2287 | 1/<1.600 |
| Banks STS | -0.04073 | -0.2362, 0.1547 | -0.413 | 0.680/<1.600 | 0.05107 | 1/<1.600 |
| Entorhinal | 0.03045 | -0.1694, 0.2303 | 0.302 | 0.763/<1.600 | 0.008584 | 1/<1.600 |
| Fusiform | -0.6106 | -0.2503, 0.1282 | -0.639 | 0.524/<1.600 | 0.1105 | 1/<1.600 |
| Inferior Parietal | 0.01447 | -0.1777, 0.2067 | 0.149 | 0.882/<1.600 | 0.08235 | 1/<1.600 |
| Inferior Temporal | -0.0257 | -0.2076, 0.1562 | -0.28 | 0.780/<1.600 | 0.1779 | 1/<1.600 |
| Lateral Occipital | 0.10339 | -0.08926, 0.2960 | 1.064 | 0.290/<1.600 | 0.07826 | 1/<1.600 |
| Lingual | -0.10601 | -0.3047, 0.09267 | -1.057 | 0.293/<1.600 | 0.01962 | 1/<1.600 |
| Middle Temporal | 0.03099 | -0.1529, 0.2149 | 0.334 | 0.739/<1.600 | 0.1599 | 1/<1.600 |
| Parahippocampal | -0.1568 | -0.3587, 0.04510 | -1.539 | 0.127/<1.600 | -0.01225 | 1/<1.600 |
| Paracentral | -0.05608 | -0.2524, 0.1403 | -0.566 | 0.573/<1.600 | 0.04264 | 1/<1.600 |
| Precentral | -0.02383 | -0.2234, 0.1758 | -0.237 | 0.813/<1.600 | 0.01059 | 1/<1.600 |
| Postcentral | 0.05025 | -0.15008, 0.2506 | 0.497 | 0.620/<1.600 | 0.003253 | 1/<1.600 |
| Precuneus | -0.0535 | -0.2371, 0.1301 | -0.578 | 0.565/<1.600 | 0.1633 | 1/<1.600 |
| **Cuneus** | **-0.2065** | **-0.3992, 0.01374** | **-2.123** | **0.036/3.074** | **0.07752** | 1/<1.600 |
| Superior Frontal | -0.07949 | -0.2697, 0.1107 | -0.8282 | 0.409/<1.600 | 0.1013 | 1/<1.600 |
| Superior Parietal | -0.02687 | -0.2167, 0.1629 | -0.281 | 0.780/<1.600 | 0.1052 | 1/<1.600 |
| Superior Temporal | 0.001539 | -0.1931, 0.1962 | 0.016 | 0.988/<1.600 | 0.05905 | 1/<1.600 |
| Parsopercularis | -0.0618 | -0.2514, 0.1271 | -0.651 | 0.516/<1.600 | 0.1106 | 1/<1.600 |
| Parsorbitalis | -0.09445 | -0.2796, 0.09066 | -1.011 | 0.314/<1.600 | 0.1491 | 1/<1.600 |
| Parstriangularis | -0.11975 | -0.3012, 0.06172 | -1.308 | 0.194/<1.600 | 0.1822 | 1/<1.600 |
| Pericalcarine | -0.18801 | -0.3871, 0.01108 | -1.871 | 0.0639/2.09 | 0.01562 | 1/<1.600 |
| Supramarginal | 0.06112 | -0.1407, 0.2629 | 0.6 | 0.550/<1.600 | -0.01146 | 1/<1.600 |
| ACC Caudal | 0.02334 | -0.1778, 0.2245 | 0.23 | 0.819/<1.600 | -0.00467 | 1/<1.600 |
| ACC Rostral | -0.11317 | -0.3011, 0.07472 | -1.194 | 0.235/<1.600 | 0.1233 | 1/<1.600 |
| Insula | -0.16697 | -0.3623, 0.02838 | -1.694 | 0.0931/1.66 | 0.05231 | 1/<1.600 |
| OFC Lateral | -0.07587 | -0.2708, 0.1190 | -0.771 | 0.442/<1.600 | 0.05643 | 1/<1.600 |
| OFC Medial | -0.09363 | -0.27705, 0.0898 | -1.012 | 0.314/<1.600 | 0.1645 | 1/<1.600 |
| Transverse Temporal | 0.05678 | -0.1376, 0.2512 | 0.579 | 0.564/<1.600 | 0.06156 | 1/<1.600 |
| Isthmus Cingulate | -0.01814 | -0.2172, 0.1809 | -0.181 | 0.857/<1.600 | 0.01573 | 1/<1.600 |
| Temporal Pole | 0.15977 | -0.03197, 0.3515 | 1.651 | 0.102/<1.600 | 0.08693 | 1/<1.600 |
| Frontal Pole | -0.13226 | -0.3199, 0.05541 | -1.397 | 0.165/<1.600 | 0.1253 | 1/<1.600 |
| Mean Thickness | -0.0663 | -0.2509, 0.1183 | -0.712 | 0.478/<1.600 | 0.1536 | 1/<1.600 |
| PCC | -0.09297 | -0.2928, 0.1068 | -0.922 | 0.359/<1.600 | 0.008641 | 1/<1.600 |

Footnotes: Regions (in mm^3^) in bold indicate a significant association between brain volume/surface area/cortical thickness and AQ-10 scores. All tests are non-parametric.

[Abbreviations: ACC – Anterior Cingulate Cortex; CB – Cerebellum; CC – Cingulate Cortex; CSF – Cerebrospinal Fluid; eTIV – estimated Total Intracranial Volume; GM – Grey Matter; HC – Healthy Control; MF – Medial Frontal; OFC – Orbitofrontal Cortex; PCC – Posterior Cingulate Cortex; STS – Superior Temporal Sulcus; Subcort – Subcortical; WM – White Matter; WR – Weight Restored]


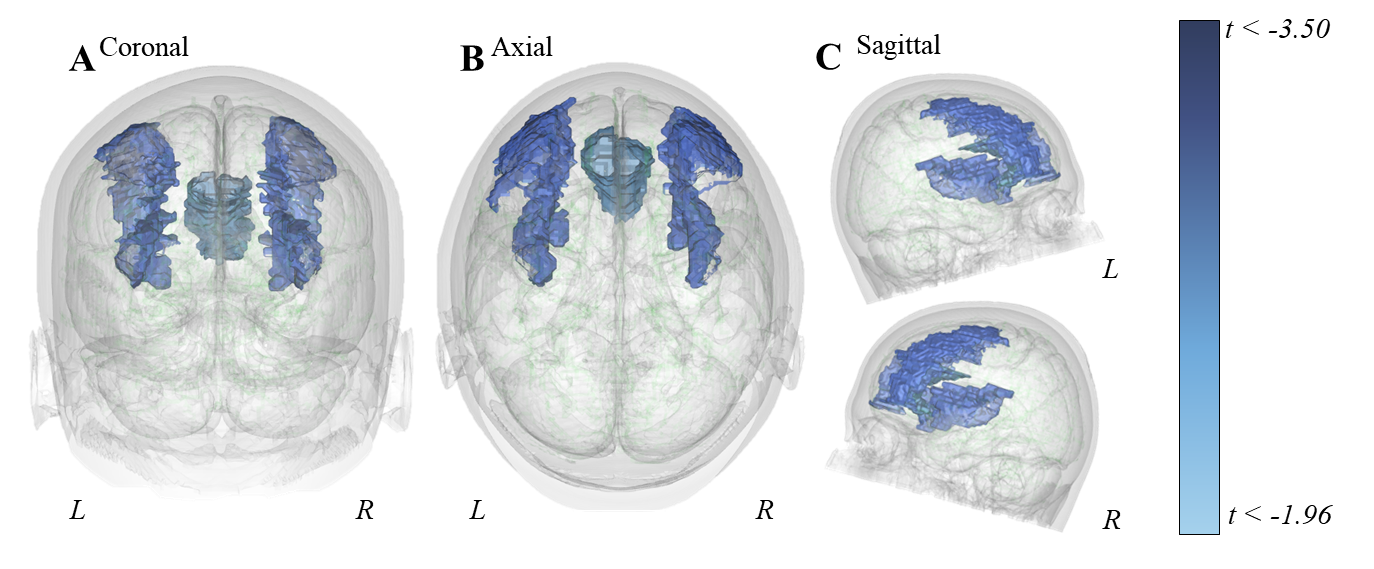


**Supplementary Figure 2.** Exploratory uncorrected associations between brain volume and AQ-10 scores in those with AAN (n=68) and those who are WR (n=48) in coronal (A), axial (B) and sagittal (C) orientations. Findings delineate a negative correlation within the mid-anterior cingulate cortex relative to increase in ADOS-2 scores [Abbreviations: L – Left; R – Right].

| **Supplementary Table 3. A-priori differences in associations between structural parameters and AQ-10 scores within the ac-AN (n=68) and WR (n=48) groups** | | | | | | | | | | |
| --- | --- | --- | --- | --- | --- | --- | --- | --- | --- | --- |
| **Region (Volume)** | **ac-AN Estimate** | **WR Estimate** | **ac-AN CI** | **WR CI** | **ac-AN p**  **/BF** | **WR p**  **/BF** | **ac-AN R2** | **WR R2** | **Fisher Z** | **Fisher Z p**  **/BF** |
| MF Caudal | 0.04721 | 0.2061 | -0.2113, 0.2968 | -0.09928, 0.5114 | 0.7380  /<1.600 | 0.1805  /<1.600 | 0.2293 | 0.0507 | 1.5078 | 0.1316  /<1.600 |
| MF Rostral | -0.07215 | **-0.3054** | -0.3106, 0.1663 | **-0.5838, -0.0270** | 0.5476  /<1.600 | **0.03231**  **/3.317** | 0.3209 | **0.2109** | 0.7527 | 0.4516  /<1.600 |
| ACC Caudal | -0.02826 | 0.004444 | -0.3092, 0.2527 | -0.3020, 0.3109 | 0.8413  /<1.600 | 0.9770  /<1.600 | 0.05779 | 0.04371 | **2.359** | **0.01834**  /5.016 |
| ACC Rostral | -0.1156 | -0.2174 | -0.3676, 0.1364 | -0.5094, 0.07570 | 0.3628  /<1.600 | 0.1406  /<1.600 | 0.2416 | 0.1314 | 0.8164 | 0.4143  /<1.600 |
| *CC Mid Anterior* | 0.0902 | -0.20842 | -0.2001, 0.3806 | -0.5274, 0.1106 | 0.5367  /<1.600 | 0.1944  /<1.600 | -0.00665 | -0.3603 | **3.998** | **6.39E-05**  **/>53.00** |
| *CC Anterior* | -0.05825 | 0.2635 | -0.3580 0.2415 | -0.04021, 0.5673 | 0.6990  /<1.600 | 0.0873  /1.728 | -0.7289 | 0.06061 | **7.844** | **4.00E-15**  **/>53.00** |
| OFC Lateral | -0.1771 | -0.03871 | -0.4389, 0.08475 | -0.3514, 0.2740 | 0.1810  /<1.600 | 0.8039  /<1.600 | 0.1816 | 0.004633 | **1.996** | **0.04597**  /2.598 |
| OFC Medial | -0.07812 | 0.129538 | -0.3280, 0.1717 | -0.1810, 0.4401 | 0.5340  /<1.600 | 0.4046  /<1.600 | 0.2548 | 0.01772 | **3.556** | **3.77E-04**  **/>53.00** |
| **Region (Area)** | **ac-AN Estimate** | **WR Estimate** | **ac-AN CI** | **WR CI** | **ac-AN p**  **/BF** | **WR p**  **/BF** | **ac-AN R2** | **WR R2** | **Fisher Z** | **Fisher Z p**  **/BF** |
| MF Caudal | 0.0195 | 0.10554 | -0.2540, 0.2930 | -0.1896, 0.4007 | 0.8871  /<1.600 | 0.4745  /<1.600 | 0.1071 | 0.113 | 0.05153 | 0.9589  /<1.600 |
| **MF Rostral** | 0.05498 | **-0.3447** | -0.2177, 0.3277 | **-0.6327, -0.05663** | 0.6883  /<1.600 | **0.0202**  **/4.667** | 0.1122 | **0.1553** | **3.945** | **7.98E-05**  **/>53.00** |
| ACC Caudal | -0.08154 | -0.00046 | -0.3712, 0.2081 | -0.3194, 03184 | 0.5757  /<1.600 | 0.9977  /<1.600 | -0.01182 | -0.03549 | 1.546 | 0.1221  /<1.600 |
| *ACC Rostral* | -0.06221 | -0.1943 | -0.3389, 0.2145 | -0.4863, 0.0976 | 0.6547  /<1.600 | 0.1864  /<1.600 | 0.8602 | 0.1321 | **3.522** | **4.00E-04**  **/>53.00** |
| OFC Lateral | -0.0894 | -0.1375 | -0.3657, 0.1869 | -0.4490, 0.1741 | 0.5202  /<1.600 | 0.3784  /<1.600 | 0.0886 | 0.01143 | **2.136** | **0.0327**  **/3.289** |
| OFC Medial | 0.1576 | 0.09184 | -0.1318, 0.4470 | -0.2126, 0.3963 | 0.2806  /<1.600 | 0.5459  /<1.600 | -0.00015 | 0.05631 | 1.1844 | 0.2363  /<1.600 |
| **Region (Thickness)** | **ac-AN Estimate** | **WR Estimate** | **ac-AN CI** | **WR CI** | **ac-AN p** | **WR p** | **ac-AN R2** | **WR R2** | **Fisher Z** | **Fisher Z p** |
| *MF Caudal* | -0.00206 | 0.07821 | -0.2612, 0.2570 | -0.2318, 0.3883 | 0.9874  /<1.600 | 0.6134  /<1.600 | 0.1985 | 0.02119 | **3.227** | **0.0013**  **/42.58** |
| MF Rostral | -0.1117 | -0.1374 | -0.3695, 0.1461 | -0.4131, 0.1383 | 0.3898  /<1.600 | 0.3203  /<1.600 | 0.2064 | 0.2259 | 0.138 | 0.8902  /<1.600 |
| ACC Caudal | 0.04946 | -0.0243 | -0.2417, 0.3408 | -0.3226, 0.2977 | 0.7350  /<1.600 | 0.9359  /<1.600 | -0.01265 | 0.02056 | 1.327 | 0.1845  /<1.600 |
| ACC Rostral | -0.1473 | -0.07357 | -0.4304, 0.1358 | -0.3405, 0.1934 | 0.3024  /<1.600 | 0.5810  /<1.600 | 0.04287 | 0.2745 | 1.916 | 0.05531  /2.298 |
| OFC Lateral | -0.1225 | 0.04509 | -0.4117, 0.1668 | -0.2581, 0.3482 | 0.4010  /<1.600 | 0.7655  /<1.600 | 0.000965 | 0.06411 | 1.495 | 0.1349  /<1.600 |
| OFC Medial | -0.08335 | -0.01735 | -0.3519, 0.1852 | -0.3149, 0.2802 | 0.5373  /<1.600 | 0.9069  /<1.600 | 0.1387 | 0.0896 | 0.4252 | 0.6707  /<1.600 |

Footnotes: Regions (in mm^3^) in bold indicate a significant association between brain volume/surface area/cortical thickness and AQ-10 scores, in either the ac-AN or WR groups. Italicised regions indicate a significant difference across relationships within subgroups, but no linear association between brain volume/thickness/surface area and AQ-10 scores. All tests are non-parametric.

[Abbreviations: ac-AN – Acute Anorexia Nervosa; ACC – Anterior Cingulate Cortex; BF – Bayes Factor; CC – Cingulate Cortex; MF – Medial Frontal; OFC – Orbitofrontal Cortex; WR – Weight Restored]

| **Supplementary Table 4. Exploratory differences in associations between structural parameters and AQ-10 scores within ac-AN (n=68) and WR (n=48) groups** | | | | | | | | | | | |
| --- | --- | --- | --- | --- | --- | --- | --- | --- | --- | --- | --- |
| **Region (Volume)** | **ac-AN Estimate** | **WR Estimate** | **ac-AN CI** | **WR CI** | **ac-AN p**  **/BF** | **WR p**  **/BF** | **ac-AN R2** | **WR R2** | **Fisher Z** | **Fisher Z p**  **/BF** | **Fisher Z p(Bonferroni)**  **/BF** |
| eTIV | 0.03286 | 0.06171 | -0.2503, 0.3161 | -0.2459, 0.3693 | 0.8173  /<1.600 | 0.6877  /<1.600 | 0.04245 | 0.03658 | 0.0793 | 0.9368  /<1.600 | 1.000  /<1.600 |
| GM Cortex | -0.0661 | -0.01953 | -0.3031, 0.1709 | -0.3101, 0.2711 | 0.579  /<1.600 | 0.8928  /<1.600 | 0.3294 | 0.1402 | 1.3397 | 0.1803  /<1.600 | 1.000  /<1.600 |
| GM Subcort | -0.16062 | 0.1238 | -0.4292, 0.1080 | -0.1914, 0.4390 | 0.2365  /<1.600 | 0.4324  /<1.600 | 0.1387 | -0.01151 | **2.573** | **0.010107**  /7.926 | 0.6464  /<1.600 |
| GM Total | -0.09673 | 0.03954 | -0.3348, 0.1413 | -0.2560, 0.3351 | 0.4200  /<1.600 | 0.7885  /<1.600 | 0.3234 | 0.1104 | **5.100** | **3.22E-07**  **/>53.00** | **2.06E-05**  **/>53.00** |
| WM Total | 0.1467 | -0.04708 | -0.1396, 0.4331 | -0.3636, 0.2694 | 0.3097  /<1.600 | 0.7655  /<1.600 | 0.02096 | -0.02023 | 1.490 | 0.1361  /<1.600 | 1.000  /<1.600 |
| MF Caudal | 0.04721 | 0.2061 | -0.2113, 0.2968 | -0.09928, 0.5114 | 0.7380  /<1.600 | 0.1805  /<1.600 | 0.2293 | 0.0507 | 1.508 | 0.1316  /<1.600 | 1.000  /<1.600 |
| **MF Rostral** | -0.07215 | **-0.3054** | -0.3106, 0.1663 | **-0.5838, -0.0270** | 0.5476  /<1.600 | **0.03231**  **/3.317** | 0.3209 | **0.2109** | 0.7527 | 0.4516  /<1.600 | 1.000  /<1.600 |
| BanksSTS | 0.09544 | 0.08678 | -0.1681, 0.3590 | -0.2200, 0.3936 | 0.4718  /<1.600 | 0.5712  /<1.600 | 0.1709 | 0.04163 | 1.200 | 0.2300  /<1.600 | 1.000  /<1.600 |
| **Entorhinal** | -0.02317 | **-0.2673** | -0.3180, 0.2717 | **-0.5217, -0.01294** | 0.8757  /<1.600 | **0.03989**  **/2.863** | -0.03795 | **0.3413** | **2.431** | **0.01504**  **/5.828** | 0.9626  /<1.600 |
| Fusiform | -0.1119 | 0.2142 | -0.3825, 0.1588 | -0.1058, 05341 | 0.4119  /<1.600 | 0.1839  /<1.600 | 0.2253 | -0.04223 | **3.736** | **0.000187**  **/>53.00** | **0.01962**  **/4.770** |
| Inferior Parietal | -0.05477 | 0.0122 | -0.3391, 0.2296 | -0.2918, 0.3162 | 0.7015  /<1.600 | 0.9359  /<1.600 | 0.0348 | 0.05881 | **2.249** | **0.02449**  /4.050 | 1.000  /<1.600 |
| Inferior Temporal | 0.00548 | -0.53 | -0.2630, 0.2740 | -0.3502, 0.2442 | 0.9676  /<1.600 | 0.7207  /<1.600 | 0.1392 | -0.1008 | **3.717** | **0.000201**  **/>53.00** | **0.0129**  **/6.560** |
| Lateral Occipital | 0.1217 | -0.1093 | -0.1607, 0.4040 | -0.4241, 0.2055 | 0.3923  /<1.600 | 0.4870  /<1.600 | 0.04824 | -0.00904 | 1.643 | 0.1003  /<1.600 | 1.000  /<1.600 |
| Lingual | -0.1907 | 0.005819 | -0.4852, 0.1037 | -0.3102, 0.3219 | 0.2000  /<1.600 | 0.9705  /<1.600 | -0.03536 | -0.01723 | 1.662 | 0.0965  **/1.630** | 1.000  /<1.600 |
| Middle Temporal | 0.07585 | -0.06506 | -0.1850, 0.03367 | -0.3767, 0.2466 | 0.5631  /<1.600 | 0.6757  /<1.600 | 0.1879 | 0.01102 | **2.937** | **0.003315**  **/1.944** | 0.2122  /<1.600 |
| **Parahippocampal** | **-0.2619** | 0.06026 | **-0.5190, -0.004838** | -0.2349, 0.3554 | **0.04597**  **/2.598** | 0.6824  /<1.600 | **0.04597** | 0.113 | **2.927** | **0.003427**  **/1.891** | 0.2133  /<1.600 |
| Paracentral | 0.07768 | -0.06085 | -0.1836, 0.3389 | -0.3682, 2465 | 0.5544  /<1.600 | 0.6920  /<1.600 | 0.1852 | 0.03793 | **3.391** | **0.000696**  **/>53.00** | **0.0457**  **/2.61** |
| Precentral | -0.2176 | 0.1528 | -0.4793, 0.04416 | -0.1641, 0.4697 | 0.1016  /<1.600 | 0.3360  /<1.600 | 0.1821 | -0.02235 | **3.128** | **0.001762**  **/32.92** | 0.1128  /<1.600 |
| Postcentral | -0.02159 | 0.1441 | -0.2958, 0.2526 | -0.1681, 0.4563 | 0.8755  /<1.600 | 0.3570  /<1.600 | 0.1022 | 0.007546 | **2.157** | **0.03070**  /3.440 | 1.000  /<1.600 |
| Precuneus | -0.1268 | 0.06861 | -0.3756, 0.1220 | -0.2478, 0.3850 | 0.3120  /<1.600 | 0.6639  /<1.600 | 0.261 | -0.01921 | **3.627** | **0.000286**  **/>53.00** | **0.01833**  **/5.018** |
| Cuneus | -0.2534 | -0.1061 | -0.5392, 0.0325 | -0.4321, 0.2198 | 0.0813  /1.803 | 0.5147  /<1.600 | 0.02449 | -0.08184 | 0.7039 | 0.4815  /<1.600 | 1.000  /<1.600 |
| Superior Frontal | 0.05334 | -0.06993 | -0.1920, 0.2987 | -0.3668, 0.2270 | 0.6650  /<1.600 | 0.6370  /<1.600 | 0.2815 | 0.1024 | **4.575** | **1.96E-06**  **/>53.00** | **0.000125**  **/>53.00** |
| Superior Parietal | -0.1538 | 0.004563 | -0.4198, 0.1122 | -0.3081, 0.3172 | 0.2522  /<1.600 | 0.9766  /<1.600 | 0.155 | 0.00482 | **2.505** | **0.01226**  **/6.817** | 0.7846  /<1.600 |
| Superior Temporal | 0.05151 | -0.1024 | -0.2164, 0.3195 | -0.3821, 0.1774 | 0.7021  /<1.600 | 0.4644  /<1.600 | 0.1427 | 0.2031 | **4.553** | **5.28E-06**  **/>53.00** | **0.000398**  **/>53.00** |
| Brainstem | 0.01767 | 0.005106 | -0.2777, 0.3131 | -0.3066, 0.3168 | 0.9052  /<1.600 | 0.9738  /<1.600 | -0.4171 | 0.01085 | **3.422** | **0.000622**  **/>53.00** | **0.03981**  **/2.867** |
| **CSF** | **-0.47197** | 0.1064 | **-0.7388, -0.2051** | -0.2170, 0.4298 | **0.000777**  **/>53.00** | 0.5103  /<1.600 | **0.1497** | -0.06491 | **3.448** | **0.000565**  **/>53.00** | **0.03613**  **/3.066** |
| Ventral DC | -0.03627 | 0.1531 | -0.3270, 0.2545 | -0.1622, 0.4685 | 0.8040  /<1.600 | 0.3327  /<1.600 | -0.00925 | -0.01251 | 1.077 | 0.2817  /<1.600 | 1.000  /<1.600 |
| Vessel | 0.12156 | -0.09209 | -0.1711, 0.4142 | -0.4090, 0.2248 | 0.4096  /<1.600 | 0.5607  /<1.600 | -0.02268 | -0.02238 | 1.560 | 0.1188  /<1.600 | 1.000  /<1.600 |
| Choroid Plexus | -0.2568 | 0.1226 | -0.5392, 0.02552 | -0.1849, 0.4301 | 0.07385  /1.912 | 0.4255  /<1.600 | 0.04816 | 0.03724 | **2.158** | **0.03092**  **/3.422** | 1.000  /<1.600 |
| Supratentorial | 0.01383 | -0.01008 | -0.2638, 0.2914 | -0.3257, 0.3056 | 0.9210  /<1.600 | 0.9490  /<1.600 | 0.0798 | -0.0144 | **2.119** | **0.03407**  **/3.195** | 1.000  /<1.600 |
| Supratentorial No Vent | 0.02717 | -0.02343 | -0.2509, 0.3052 | -0.3374, 0.2906 | 0.8458  /<1.600 | 0.8810  /<1.600 | 0.7697 | -0.00403 | **7.362** | **1.82E-13**  **/>53.00** | **1.17E-11**  **/>53.00** |
| Parsopercularis | -0.1839 | 0.06843 | -0.4655, 0.9778 | -0.2424, 0.3793 | 0.1967  /<1.600 | 0.6591  /<1.600 | 0.05287 | 0.01631 | 1.869 | 0.06156  /2.144 | 1.000  /<1.600 |
| Parsorbitalis | -0.0773 | -0.08531 | -0.3471, 0.1925 | -0.3851, 0.2145 | 0.5687  /<1.600 | 0.5689  /<1.600 | 0.1309 | 0.08473 | 0.4084 | 0.6830  /<1.600 | 1.000  /<1.600 |
| Parstriangularis | -0.03244 | -0.145 | -0.3158, 0.2509 | -0.4408, 0.1508 | 0.8197  /<1.600 | 0.3282  /<1.600 | 0.04156 | 0.109 | 0.7025 | 0.4823  /<1.600 | 1.000  /<1.600 |
| Pericalcarine | -0.2218 | -0.1091 | -0.5129, 0.06923 | -0.4290, 0.2107 | 0.1330  /<1.600 | 0.4950  /<1.600 | -0.0115 | -0.04174 | 0.5130 | 0.6076  /<1.600 | 1.000  /<1.600 |
| Supramarginal | -0.14 | 0.05454 | -0.3953, 0.1153 | -0.2681, 0.3772 | 0.2770  /<1.600 | 0.7347  /<1.600 | 0.2218 | -0.05975 | **3.923** | **8.74E-05**  **/>53.00** | **0.00594**  **/12.49** |
| ACC Caudal | -0.02826 | 0.004444 | -0.3092, 0.2527 | -0.3020, 0.3109 | 0.8413  /<1.600 | 0.9770  /<1.600 | 0.05779 | 0.04371 | **2.359** | **0.01834**  /5.016 | 1.000  /<1.600 |
| ACC Rostral | -0.1156 | -0.2174 | -0.3676, 0.1364 | -0.5094, 0.07570 | 0.3628  /<1.600 | 0.1406  /<1.600 | 0.2416 | 0.1314 | 0.8164 | 0.4143  /<1.600 | 1.000  /<1.600 |
| **Insula** | **-0.2984** | -0.1384 | **-0.5646, -0.03208** | -0.4480, 0.1712 | **0.02870**  **/3.610** | 0.3722  /<1.600 | **0.1535** | 0.02397 | 1.330 | 0.1837  /<1.600 | 1.000  /<1.600 |
| OFC Lateral | -0.1771 | -0.03871 | -0.4389, 0.08475 | -0.3514, 0.2740 | 0.1810  /<1.600 | 0.8039  /<1.600 | 0.1816 | 0.004633 | **1.996** | **0.04597**  /2.598 | 1.000  /<1.600 |
| OFC Medial | -0.07812 | 0.129538 | -0.3280, 0.1717 | -0.1810, 0.4401 | 0.5340  /<1.600 | 0.4046  /<1.600 | 0.2548 | 0.01772 | **3.556** | **3.77E-04**  **/>53.00** | 0.02413  /1.208 |
| Frontal Pole | -0.03569 | -0.1172 | -0.3249, 0.2535 | -0.3861, 0.1516 | 0.8060  /<1.600 | 0.3839  /<1.600 | 0.001639 | 0.2638 | **2.718** | **0.006564**  **/11.15** | 0.4201  /<1.600 |
| Temporal Pole | -0.1212 | -0.00042 | -0.4132, 0.1710 | -0.3151, 0.3142 | 0.4102  /<1.600 | 0.9979  /<1.600 | -0.0187 | -0.00798 | 0.2478 | 0.8043  /<1.600 | 1.000  /<1.600 |
| Transverse Temporal | -0.1126 | -0.07182 | -0.3958, 0.1705 | -0.3893, 0.2456 | 0.4297  /<1.600 | 0.6503  /<1.600 | 0.0427 | -0.02626 | 0.2381 | 0.8118  /<1.600 | 1.000  /<1.600 |
| Isthmus Cingulate | -0.1922 | -0.01881 | -0.4774, 0.09311 | -0.3196, 0.2816 | 0.1830  /<1.600 | 0.9002  /<1.600 | 0.02834 | 0.07895 | 0.6125 | 0.5402  /<1.600 | 1.000  /<1.600 |
| CC Posterior | 0.001322 | -0.1026 | -0.2883, 0.2910 | -0.4191, 0.2140 | 0.9927  /<1.600 | 0.5168  /<1.600 | -0.00179 | -0.02029 | 0.9579 | 0.3381  /<1.600 | 1.000  /<1.600 |
| CC Mid Posterior | 0.02898 | -0.1883 | -0.2685, 0.3264 | -0.5057, 0.1291 | 0.8463  /<1.600 | 0.2379  /<1.600 | -0.05682 | -0.02568 | **2.087** | **0.0369**  /3.022 | 1.000  /<1.600 |
| CC Central | 0.2593 | -0.1915 | -0.03020, 0.5488 | -0.5097, 0.1268 | 0.0783  /1.845 | 0.2315  /<1.600 | -0.00064 | -0.03135 | 1.054 | 0.2921  /<1.600 | 1.000  /<1.600 |
| CC Mid Anterior | 0.0902 | -0.20842 | -0.2001, 0.3806 | -0.5274, 0.1106 | 0.5367  /<1.600 | 0.1944  /<1.600 | -0.00665 | -0.3603 | **3.998** | **6.39E-05**  **/>53.00** | **0.00409**  **/16.36** |
| CC Anterior | -0.05825 | 0.2635 | -0.3580 0.2415 | -0.04021, 0.5673 | 0.6990  /<1.600 | 0.0873  /1.728 | -0.7289 | 0.06061 | **7.844** | **4.00E-15**  **/>53.00** | **2.56E-13**  **/>53.00** |
| Amygdala | 0.04578 | 0.03438 | -0.2449, 0.3364 | -0.2738, 0.3426 | 0.7540  /<1.600 | 0.8230  /<1.600 | -0.00869 | 0.03282 | 0.4625 | 0.6437  /<1.600 | 1.000  /<1.600 |
| Hippocampus | -0.1437 | 0.1866 | -0.4186, 0.1313 | -0.1094, 0.4826 | 0.3003  /<1.600 | 0.2103  /<1.600 | 0.0975 | 0.1078 | **3.424** | **6.17E-04**  **/>53.00** | **0.03949**  **/2.883** |
| CB GM | -0.0942 | 0.1781 | -0.3718, 0.1821 | -0.1263, 0.4824 | 0.4963  /<1.600 | 0.2444  /<1.600 | 0.0842 | 0.05656 | **2.791** | **0.005257**  **/1.333** | 0.3364  /<1.600 |
| CB WM | 0.07831 | 0.08812 | -0.2110, 0.3676 | -0.2313, 0.4076 | 0.5903  /<1.600 | 0.5807  /<1.600 | 0.000921 | -0.03896 | 0.8748 | 0.3817  /<1.600 | 1.000  /<1.600 |
| **Caudate** | **-0.3086** | 0.2934 | **-0.5823, -0.03491** | -0.01601, 0.6027 | **0.0277**  **/3.703** | 0.0625  /2.123 | **0.1057** | 0.02554 | **2.571** | **0.01015**  **/7.900** | 0.6496  /<1.600 |
| Putamen | -0.1652 | -0.1168 | -0.4424, 0.1120 | -0.4412, 0.2076 | 0.2380  /<1.600 | 0.4716  /<1.600 | 0.08278 | -0.07159 | **2.941** | **0.003273**  **/19.64** | 0.2095  /<1.600 |
| Pallidum | -0.1723 | 0.12 | -0.4544, 0.1098 | -0.2018, 0.4419 | 0.2267  /<1.600 | 0.4558  /<1.600 | 0.0499 | -0.05461 | **2.399** | **0.01642**  **/5.452** | 1.000  /<1.600 |
| Accumbens | 0.006922 | 0.2711 | -0.2872, 0.3011 | -0.04801, 0.5901 | 0.9626  /<1.600 | 0.09382  /1.657 | -0.03304 | -0.03675 | 0.05308 | 0.9577  /<1.600 | 1.000  /<1.600 |
| Thalamus Proper | -0.0711 | 0.03855 | -0.3388, 0.1966 | -0.2800, 0.3571 | 0.5973  /<1.600 | 0.8083  /<1.600 | 0.1445 | -0.03339 | **3.017** | **0.002556**  **/24.11** | 0.1636  /<1.600 |
| Lateral Ventricle | -0.2652 | 0.1354 | -0.5475, 0.01716 | -0.1866, 0.4574 | 0.06516  /<1.600 | 0.4010  /<1.600 | 0.4819 | -0.05585 | **5.656** | **1.55E-08**  **/>53.00** | **9.92E-07**  **/>53.00** |
| Inferior Lateral Ventricle | -0.245 | 0.01323 | -0.5312, 0.04127 | -0.3045, 0.3310 | 0.0921  /<1.6748 | 0.9334  /<1.600 | 0.02188 | -0.02785 | 1.637 | 0.1016  /<1.600 | 1.000  /<1.600 |
| Third Ventricle | -0.1954 | -0.1428 | -0.4860, 0.09516 | -0.4510, 0.1655 | 0.1837  /<1.600 | 0.3553  /<1.600 | -0.00821 | 0.03252 | 0.4718 | 0.6371  /<1.600 | 1.000  /<1.600 |
| Fourth Ventricle | -0.04444 | 0.0725 | -0.3352, 0.2462 | -0.2149, 0.3600 | 0.7610  /<1.600 | 0.6134  /<1.600 | -0.00904 | 0.159 | **2.669** | **0.007617**  **/9.902** | 0.4875  /<1.600 |
| Fifth Ventricle | N/A | N/A | N/A | N/A | N/A | N/A | N/A | N/A | N/A | N/A | N/A |
| **PCC** | **-0.3015** | -0.0057 | **-0.5561, -0.04700** | -0.3221, 0.3107 | **0.021**  **/4.535** | 0.9712  /<1.600 | **0.2263** | -0.01936 | 1.946 | 0.05166  /2.403 | 1.000  /<1.600 |
| **Region (Area)** | **ac-AN Estimate** | **WR Estimate** | **ac-AN CI** | **WR CI** | **ac-AN p**  **/BF** | **WR p**  **/BF** | **ac-AN R2** | **WR R2** | **Fisher Z** | **Fisher Z p**  **/BF** | **Fisher Z p(Bonferroni)**  **/BF** |
| MF Caudal | 0.0195 | 0.10554 | -0.2540, 0.2930 | -0.1896, 0.4007 | 0.8871  /<1.600 | 0.4745  /<1.600 | 0.1071 | 0.113 | 0.05153 | 0.9589  /<1.600 | 1.000  /<1.600 |
| **MF Rostral** | 0.05498 | **-0.3447** | -0.2177, 0.3277 | **-0.6327, -0.05663** | 0.6883  /<1.600 | **0.0202**  **/4.667** | 0.1122 | **0.1553** | **3.945** | **7.98E-05**  **/>53.00** | **0.002793**  **/22.40** |
| Banks STS | 0.09485 | 0.1343 | -0.1751, 0.3648 | -0.1806, 0.4491 | 0.4851  /<1.600 | 0.3944  /<1.600 | 0.1298 | -0.00956 | 1.439 | 0.1500  /<1.600 | 1.000  /<1.600 |
| **Entorhinal** | **-0.3105** | -0.08499 | **-0.5969, -0.02405** | -0.3817 0.2118 | **0.0341**  **/3.190** | 0.5663  /<1.600 | **0.02064** | 0.1034 | 0.9731 | 0.3305  /<1.600 | 1.000  /<1.600 |
| Fusiform | -0.727 | 0.234 | -0.3422, 0.1968 | -0.08113, 0.5491 | 0.5917  /<1.600 | 0.1415  /<1.600 | 0.1328 | -0.1091 | **3.739** | **0.000185**  **/>53.00** | **0.006461**  **/11.29** |
| Inferior Parietal | -0.08679 | -0.01919 | -0.3745, 0.2009 | -0.3391, 0.3007 | 0.5487  /<1.600 | 0.9042  /<1.600 | 0.01185 | -0.04181 | 0.5059 | 0.6129  /<1.600 | 1.000  /<1.600 |
| Inferior Temporal | -0.06861 | -0.05156 | -0.3483, 0.2111 | -0.3552, 0.2521 | 0.6256  /<1.600 | 0.7336  /<1.600 | 0.06589 | 0.06095 | **2.654** | **0.007957**  **/9.565** | 0.2785  /<1.600 |
| Lateral Occipital | -0.08672 | -0.03491 | -0.3803, 0.2068 | -0.3605, 0.2907 | 0.5570  /<1.600 | 0.8300  /<1.600 | -0.02897 | -0.07934 | **2.379** | **0.01735**  **/5.230** | 0.6073  /<1.600 |
| Lingual | -0.05131 | -0.00106 | -0.3483, 0.2457 | -0.3094, 0.3072 | 0.7311  /<1.600 | 0.9945  /<1.600 | -0.05335 | 0.03221 | **2.149** | **0.03167**  **/3.365** | 1.000  /<1.600 |
| Middle Temporal | 0.04292 | -0.1044 | -0.2352, 0.3210 | -0.4232, 0.2145 | 0.7587  /<1.600 | 0.5125  /<1.600 | 0.07666 | -0.03504 | 0.4892 | 0.6247  /<1.600 | 1.000  /<1.600 |
| Parahippocampal | -0.00686 | 0.09051 | -0.2783, 0.2646 | -0.2255, 0.4065 | 0.9599  /<1.600 | 0.5663  /<1.600 | 0.1205 | -0.01692 | **2.542** | **0.01106**  **/7.384** | 0.3871  /<1.600 |
| Paracentral | 0.06186 | -0.108 | -0.2060, 0.3927 | -0.4043, 0.1884 | 0.6459  /<1.600 | 0.46633  /<1.600 | 0.1435 | 0.1058 | **0.3153** | 0.7525  /<1.600 | 1.000  /<1.600 |
| Precentral | -0.2272 | 0.0716 | -0.4932, 0.03874 | -0.2419, 0.3851 | 0.09267  /1.669 | 0.6472  /<1.600 | 0.1554 | -0.00077 | **2.292** | **0.0219**  **/4.396** | 0.7665  /<1.600 |
| Postcentral | -0.05784 | 0.1051 | -0.3236, 0.2079 | -0.2080, 0.4182 | 0.6650  /<1.600 | 0.5018  /<1.600 | 0.1569 | 0.00182 | **2.381** | **0.01727**  **/5.248** | 0.6045  /<1.600 |
| Precuneus | 0.002133 | -0.04363 | -0.2750, 0.2792 | -0.3591, 0.2718 | 0.9878  /<1.600 | 0.7820  /<1.600 | 0.08327 | -0.01338 | 0.9324 | 0.3511  /<1.600 | 1.000  /<1.600 |
| Cuneus | -0.1348 | -0.1051 | -0.4294, 0.1599 | -0.4265, 0.2164 | 0.3640  /<1.600 | 0.5130  /<1.600 | -0.03633 | -0.05201 | **2.192** | **0.02837**  **/3.640** | 0.993  /<1.600 |
| Superior Frontal | 0.07265 | -0.1079 | -0.2065, 0.3518 | -0.4219, 0.2060 | 0.6048  /<1.600 | 0.4917  /<1.600 | 0.06936 | -0.00352 | 1.084 | 0.2781  /<1.600 | 1.000  /<1.600 |
| Superior Parietal | -0.1541 | -0.00615 | -0.4213, 0.1131 | -0.3135, 0.3012 | 0.2534  /<1.600 | 0.9680  /<1.600 | 0.1478 | 0.03795 | **3.107** | **0.001888**  **/31.066** | 0.06608  /2.049 |
| Superior Temporal | -0.06296 | -0.1428 | -0.3404, 0.2144 | -0.4347, 0.1491 | 0.6517  /<1.600 | 0.3292  /<1.600 | 0.08124 | 0.1325 | **3.479** | **0.000504**  **/>53.00** | **0.01762**  **/5.170** |
| Parsopercularis | -0.128 | 0.07511 | -0.4179, 0.1620 | -0.2331, 0.3833 | 0.3810  /<1.600 | 0.6255  /<1.600 | -0.00354 | 0.03259 | 1.248 | 0.2119  /<1.600 | 1.000  /<1.600 |
| Parsorbitalis | -0.1467 | 0.03017 | -0.4254, 0.1320 | -0.2859, 0.3462 | 0.2968  /<1.600 | 0.8482  /<1.600 | 0.07246 | -0.0171 | **2.101** | **0.0356**  **/3.098** | 1.000  /<1.600 |
| Parstriangularis | -0.06824 | -0.1267 | -0.3643, 0.2278 | -0.4316, 0.1782 | 0.6466  /<1.600 | 0.4065  /<1.600 | -0.04669 | 0.0533 | **2.344** | **0.0191**  **/4.866** | 0.6685  /<1.600 |
| Pericalcarine | -0.1497 | -0.00242 | -0.04458, 0.1464 | -0.3231, 0.3182 | 0.3160  /<1.600 | 0.9879  /<1.600 | -0.0469 | -0.04695 | **2.270** | **0.0232**  **/4.213** | 0.8120  /<1.600 |
| Supramarginal | -0.2012 | 0.05768 | -0.4512, 0.04876 | -0.2625, 0.3778 | 0.1127  /<1.600 | 0.7180  /<1.600 | 0.254 | -0.04361 | **3.953** | **7.72E-05**  **/>53.00** | **0.02702**  **/3.770** |
| ACC Caudal | -0.08154 | -0.00046 | -0.3712, 0.2081 | -0.3194, 03184 | 0.5757  /<1.600 | 0.9977  /<1.600 | -0.01182 | -0.03549 | 1.546 | 0.1221  /<1.600 | 1.000  /<1.600 |
| ACC Rostral | -0.06221 | -0.1943 | -0.3389, 0.2145 | -0.4863, 0.0976 | 0.6547  /<1.600 | 0.1864  /<1.600 | 0.8602 | 0.1321 | **3.522** | **4.00E-04**  **/>53.00** | **0.01400**  **/6.156** |
| Insula | -0.1413 | 0.006376 | -0.4234, 0.1408 | -0.3079, 0.3206 | 0.3205  /<1.600 | 0.9675  /<1.600 | 0.05006 | -0.0056 | 1.560 | 0.1187  /<1.600 | 1.000  /<1.600 |
| OFC Lateral | -0.0894 | -0.1375 | -0.3657, 0.1869 | -0.4490, 0.1741 | 0.5202  /<1.600 | 0.3784  /<1.600 | 0.0886 | 0.01143 | **2.136** | **0.0327**  **/3.289** | 1.000  /<1.600 |
| OFC Medial | 0.1576 | 0.09184 | -0.1318, 0.4470 | -0.2126, 0.3963 | 0.2806  /<1.600 | 0.5459  /<1.600 | -0.00015 | 0.05631 | 1.184 | 0.2363  /<1.600 | 1.000  /<1.600 |
| Transverse Temporal | -0.08554 | -0.07911 | -0.3779, 0.2068 | -0.3588, 0.2006 | 0.5608  /<1.600 | 0.5712  /<1.600 | -0.0205 | 0.2033 | **3.249** | **0.0012**  **/45.58** | **0.04200**  **/2.763** |
| Isthmus Cingulate | -0.2237 | -0.05057 | -0.5152, 0.06782 | -0.3572, 0.2560 | 0.1300  /<1.600 | 0.7409  /<1.600 | -0.01446 | 0.04277 | 1.705 | 0.0882  /1.718 | 1.000  /<1.600 |
| Temporal Pole | -0.186 | -0.1506 | -0.4732, 0.1013 | -0.4560, 0.1549 | 0.2004  /<1.600 | 0.3255  /<1.600 | 0.01493 | 0.05007 | 1.807 | 0.0708  /1.962 | 1.000  /<1.600 |
| Frontal Pole | 0.02575 | -0.09057 | -0.2611, 0.3126 | -0.3708, 0.1897 | 0.8582  /<1.600 | 0.5178  /<1.600 | 0.01766 | 0.2004 | 1.795 | 0.0727  /1.930 | 1.000  /<1.600 |
| WM Surface Area | -0.1426 | -0.0276 | -0.4020, 0.1168 | -0.3343, 0.2791 | 0.2760  /<1.600 | 0.8568  /<1.600 | 0.1966 | 0.0421 | **3.530** | **4.00E-04**  **/>53.00** | **0.01400**  **/6.156** |
| PCC | -0.2011 | 0.06262 | -0.4824, 0.0831 | -0.2559, 0.3811 | 0.1582  /<1.600 | 0.6935  /<1.600 | 0.05476 | -0.03266 | **2.172** | **0.0299**  **/3.505** | 1.000  /<1.600 |
| **Region (Thickness)** | **ac-AN Estimate** | **WR Estimate** | **ac-AN CI** | **WR CI** | **ac-AN p**  **/BF** | **WR p**  **/BF** | **ac-AN R2** | **WR R2** | **Fisher Z** | **Fisher Z p**  **/BF** | **Fisher Z p(Bonferroni)**  **/BF** |
| MF Caudal | -0.00206 | 0.07821 | -0.2612, 0.2570 | -0.2318, 0.3883 | 0.9874  /<1.600 | 0.6134  /<1.600 | 0.1985 | 0.02119 | **3.227** | **0.0013**  **/42.58** | **0.0455**  **/2.617** |
| MF Rostral | -0.1117 | -0.1374 | -0.3695, 0.1461 | -0.4131, 0.1383 | 0.3898  /<1.600 | 0.3203  /<1.600 | 0.2064 | 0.2259 | 0.138 | 0.8902  /<1.600 | 1.000  /<1.600 |
| Banks STS | -0.135 | 0.1398 | -0.4012, 0.1402 | -0.1508, 0.4304 | 0.3388  /<1.600 | 0.3371  /<1.600 | 0.1252 | 0.1402 | **3.937** | **1.00E-04**  **/>53.00** | **0.0035**  **/18.57** |
| Entorhinal | 0.1389 | -0.1041 | -0.1467, 0.4246 | -0.4319, 0.2236 | 0.3346  /<1.600 | 0.5249  /<1.600 | 0.02595 | -0.09384 | **2.47** | **0.0135**  /6.330 | 0.4725  /<1.600 |
| Fusiform | -0.0172 | -0.04978 | -0.3012, 0.2675 | -0.3276, 0.2281 | 0.9040  /<1.600 | 0.7195  /<1.600 | 0.03212 | 0.2141 | 1.648 | 0.0994  /1.603 | 1.000  /<1.600 |
| Inferior Parietal | 0.02454 | 0.09127 | -0.2528, 0.3018 | -0.2059, 0.3884 | 0.8601  /<1.600 | 0.5387  /<1.600 | 0.08191 | 0.1009 | 0.1785 | 0.8584  /<1.600 | 1.000  /<1.600 |
| Inferior Temporal | 0.151 | -0.0776 | -0.1192, 0.4212 | -0.3439, 0.1887 | 0.2682  /<1.600 | 0.5597  /<1.600 | 0.1285 | 0.2777 | **4.956** | **7.20E-07**  **/>53.00** | **2.52E-05**  **/>53.00** |
| **Lateral Occipital** | **0.3376** | -0.0881 | **0.06147, 0.6137** | -0.3677, 0.1915 | **0.01738**  **/5.223** | 0.5282  /<1.600 | **0.08981** | 0.204 | **4.104** | **4.05E-05**  **/>53.00** | **0.001417**  **/39.58** |
| Lingual | -0.08496 | -0.03202 | -0.3829, 0.2130 | -0.3331, 0.2691 | 0.5710  /<1.600 | 0.8311  /<1.600 | -0.05987 | 0.07673 | **2.755** | **0.005877**  **/12.19** | 0.2057  /<1.600 |
| Middle Temporal | 0.1808 | 0.03106 | -0.0867, 0.4483 | -0.3152, 0.2530 | 0.1817  /<1.600 | 0.82644  /<1.600 | 0.1456 | 0.1782 | 0.2495 | 0.8030  /<1.600 | 1.000  /<1.600 |
| Parahippocampal | **-0.2981** | 0.04715 | **-0.5832, -0.01300** | -0.2760, 0.3702 | **0.04070**  **/2.823** | 0.7698  /<1.600 | **0.02939** | -0.06301 | **2.216** | **0.02670**  **/3.802** | 0.9345  /<1.600 |
| Paracentral | -0.02092 | -0.11368 | -0.3109, 0.2691 | -0.3970, 0.1697 | 0.8860  /<1.600 | 0.42266  /<1.600 | -0.00414 | 0.1826 | **2.022** | **0.04315**  **/2.713** | 1.000  /<1.600 |
| Precentral | -0.1877 | 0.09672 | -0.4749, 0.09958 | -0.1840, 0.3774 | 0.1960  /<1.600 | 0.4907  /<1.600 | 0.01485 | 0.1977 | **3.096** | **0.001959**  **/30.117** | 0.06857  /2.002 |
| Postcentral | 0.05444 | 0.09086 | -0.2403, 0.3492 | -0.2123, 0.3940 | 0.7132  /<1.600 | 0.5485  /<1.600 | -0.0374 | 0.06414 | 0.325 | 0.7451  /<1.600 | 1.000  /<1.600 |
| Precuneus | -0.07198 | 0.001345 | -0.3412, 0.1973 | -0.2888, 0.2915 | 0.5951  /<1.600 | 0.9926  /<1.600 | 0.1341 | 0.1426 | **4.029** | **5.60E-05**  **/>53.00** | **0.00196**  /30.10 |
| Cuneus | -0.1943 | -0.2174 | -0.4742, 0.08557 | -0.5167, 0.8189 | 0.1702  /<1.600 | 0.1501  /<1.600 | 0.06467 | 0.08789 | 0.2353 | 0.8139  /<1.600 | 1.000  /<1.600 |
| Superior Frontal | -0.01276 | -0.1118 | -0.2960, 0.2705 | -0.3940, 0.1704 | 0.9285  /<1.600 | 0.4285  /<1.600 | 0.04229 | 0.1892 | 1.3273 | 0.1844  /<1.600 | 1.000  /<1.600 |
| Superior Parietal | 0.001101 | -0.20442 | -0.2722, 0.2744 | -0.3263, 0.2274 | 0.9936  /<1.600 | 0.8711  /<1.600 | 0.1084 | 0.07223 | **3.184** | **0.001451**  **/38.79** | 0.05079  /2.431 |
| Superior Temporal | 0.0955 | 0.01016 | -0.1897, 0.3807 | -0.2797, 0.3001 | 0.5060  /<1.600 | 0.94396  /<1.600 | 0.02903 | 0.1443 | 1.175 | 0.24002  /<1.600 | 1.000  /<1.600 |
| Parsopercularis | -0.02028 | 0.006908 | -0.3019, 0.2613 | -0.2539, 0.2677 | 0.8860  /<1.600 | 0.9576  /<1.600 | 0.053 | 0.3073 | **4.43** | **9.43E-06**  **/>53.00** | **0.00033**  **/>53.00** |
| Parsorbitalis | 0.01432 | -0.1445 | -0.2574, 0.2860 | -0.4340, 0.1450 | 0.9164  /<1.600 | 0.3197  /<1.600 | 0.1187 | 0.1464 | **3.931** | **8.45E-05**  **/>53.00** | **0.002957**  /21.36 |
| Parstriangularis | -0.03849 | -0.1611 | -0.3110, 0.2340 | -0.4305, 0.1083 | 0.7786  /<1.600 | 0.2343  /<1.600 | 0.1136 | 0.261 | 1.099 | 0.2717  /<1.600 | 1.000  /<1.600 |
| Pericalcarine | -0.1615 | -0.1673 | -0.4485, 0.1255 | -0.4856, 0.1511 | 0.2650  /<1.600 | 0.2950  /<1.600 | 0.01629 | -0.03171 | 0.2664 | 0.7899  /<1.600 | 1.000  /<1.600 |
| Supramarginal | 0.05572 | 0.09357 | -0.2435, 0.3550 | -0.2161, 0.4033 | 0.7110  /<1.600 | 0.5453  /<1.600 | -0.06914 | 0.02327 | 0.5957 | 0.5514  /<1.600 | 1.000  /<1.600 |
| ACC Caudal | 0.04946 | -0.0243 | -0.2417, 0.3408 | -0.3226, 0.2977 | 0.7350  /<1.600 | 0.9359  /<1.600 | -0.01265 | 0.02056 | 1.327 | 0.1845  /<1.600 | 1.000  /<1.600 |
| ACC Rostral | -0.1473 | -0.07357 | -0.4304, 0.1358 | -0.3405, 0.1934 | 0.3024  /<1.600 | 0.5810  /<1.600 | 0.04287 | 0.2745 | 1.916 | 0.05531  /2.298 | 1.000  /<1.600 |
| Insula | -0.2395 | 0.01122 | -0.5281, 0.04922 | -0.3021, 0.3245 | 0.1020  /<1.600 | 0.9427  /<1.600 | 0.005087 | 0.000404 | 0.472 | 0.6369  /<1.600 | 1.000  /<1.600 |
| OFC Lateral | -0.1225 | 0.04509 | -0.4117, 0.1668 | -0.2581, 0.3482 | 0.4010  /<1.600 | 0.7655  /<1.600 | 0.000965 | 0.06411 | 1.495 | 0.1349  /<1.600 | 1.000  /<1.600 |
| OFC Medial | -0.08335 | -0.01735 | -0.3519, 0.1852 | -0.3149, 0.2802 | 0.5373  /<1.600 | 0.9069  /<1.600 | 0.1387 | 0.0896 | 0.4252 | 0.6707  /<1.600 | 1.000  /<1.600 |
| Transverse Temporal | 0.01935 | 0.04886 | -0.2692, 0.3079 | -0.2378, 0.3355 | 0.8938  /<1.600 | 0.7326  /<1.600 | 0.00618 | 0.1632 | 1.803 | 0.0714  /1.950 | 1.000  /<1.600 |
| Isthmus Cingulate | 0.007657 | -0.07172 | -0.2773, 0.2926 | -0.3734, 0.2300 | 0.9570  /<1.600 | 0.6339  /<1.600 | 0.03089 | 0.07341 | 0.5171 | 0.6051  /<1.600 | 1.000  /<1.600 |
| Temporal Pole | 0.2177 | 0.1396 | -0.05717, 0.4926 | -0.1644, 0.4437 | 0.1185  /<1.600 | 0.3593  /<1.600 | 0.09787 | 0.05848 | 0.397 | 0.6914  /<1.600 | 1.000  /<1.600 |
| Frontal Pole | -0.1038 | -0.1874 | -0.3862, 0.1786 | -0.4662, 0.09146 | 0.4650  /<1.600 | 0.1823  /<1.600 | 0.04754 | 0.2083 | 1.398 | 0.1620  /<1.600 | 1.000  /<1.600 |
| Mean Thickness | -0.00595 | -0.06388 | -0.2751, 0.26322 | -0.3384, 0.2107 | 0.9649  /<1.600 | 0.6411  /<1.600 | 0.1349 | 0.2326 | 0.7254 | 0.4682  /<1.600 | 1.000  /<1.600 |
| PCC | -0.04113 | -0.1563 | -0.3354, 0.2431 | -0.4753, 0.1627 | 0.7810  /<1.600 | 0.3280  /<1.600 | -0.03375 | -0.0363 | 0.0364 | 0.9710  /<1.600 | 1.000  /<1.600 |

Footnotes: Regions (in mm^3^) in bold indicate a significant association between brain volume/surface area/cortical thickness and AQ-10 scores, in either the ac-AN or WR groups. Italicised regions indicate a significant difference across relationships within subgroups, but no linear association between brain volume/thickness/surface area and AQ-10 scores. All tests are non-parametric.

[Abbreviations: ac-AN – Acute Anorexia Nervosa; ACC – Anterior Cingulate Cortex; BF – Bayes Factor; CB – Cerebellum; CC – Cingulate Cortex; CSF – Cerebrospinal Fluid; eTIV – estimated Total Intracranial Volume; GM – Grey Matter; MF – Medial Frontal; OFC – Orbitofrontal Cortex; PCC – Posterior Cingulate Cortex; STS – Superior Temporal Sulcus; Subcort – Subcortical; WM – White Matter; WR – Weight Restored]

*3.4 Volumetric Structure and AQ-10 Scores Predicting Future Social Behaviour*

In the exploratory TAS-20 random forest regression (RFR), the 10 most important variables consisted of SAS and TAS-20 scores in the first research timepoint (33.7% and 29.3% respectively), OCI score (6.1%), WSAS score (5.0%), HADS score (4.8%), middle temporal thickness (4.3%), baseline BMI (3.3%), eating concern subscale scores on the EDE-Q (3.1%), NART scores (3.0%) and total scores on the EDE-Q (2.7%). For the exploratory SAS RFR model, the top 10 variables explaining the variation of score prediction consisted of SAS scores in the first research timepoint (77.5%), middle temporal volume (5.2%), TAS-20 scores in the first research timepoint (3.7%%), frontal pole surface area (3.6%), isthmus cingulate volume (2.5%), parsopercularis surface area (2.4%), precentral thickness (2.3%), fusiform thickness (2.1%), volume of the parsopercularis (2.0%), and thickness of the banks superior temporal sulcus (STS) (1.8%).

| **Supplementary Table 4.** Performance of a-priori RFR models to predict future scores on the TAS-20 and SAS | | | | | | |
| --- | --- | --- | --- | --- | --- | --- |
| **Model** | **OOB Error (Training)** | **% Variance Explained (Training)** | **MSR (Test)** | **MSE (Test)** | **MAE (Test)** | **R^2^ (Test)** |
| A-Priori TAS-20 | 11.21 | 46.72 | 123.80 | 137.82 | 9.04 | 0.37 |
| A-Priori SAS | 6.57 | 38.03 | 42.28 | 42.80 | 5.20 | 0.29 |

[Abbreviations: MAE – Mean Absolute Error; MSE – Mean Squared Error; MSR – Mean Squared Residuals; OOB – Out of Bag Error; RFR – Random Forest Regression; SAS – Social Anhedonia Scale; TAS – Toronto Alexithymia Scale]

| **Supplementary Table 5.** Performance of exploratory RFR models to predict future scores on the TAS-20 and SAS | | | | | | |
| --- | --- | --- | --- | --- | --- | --- |
| **Model** | **OOB Error (Training)** | **% Variance Explained (Training)** | **MSR (Test)** | **MSE (Test)** | **MAE (Test)** | **R^2 (Test)** |
| Exploratory TAS-20 | 11.39 | 43.81 | 127.58 | 152.88 | 9.63 | 0.33 |
| Exploratory SAS | 6.43 | 40.66 | 40.90 | 42.43 | 4.80 | 0.31 |

[Abbreviations: MAE – Mean Absolute Error; MSE – Mean Squared Error; MSR – Mean Squared Residuals; OOB – Out of Bag Error; RFR – Random Forest Regression; SAS – Social Anhedonia Scale; TAS – Toronto Alexithymia Scale]


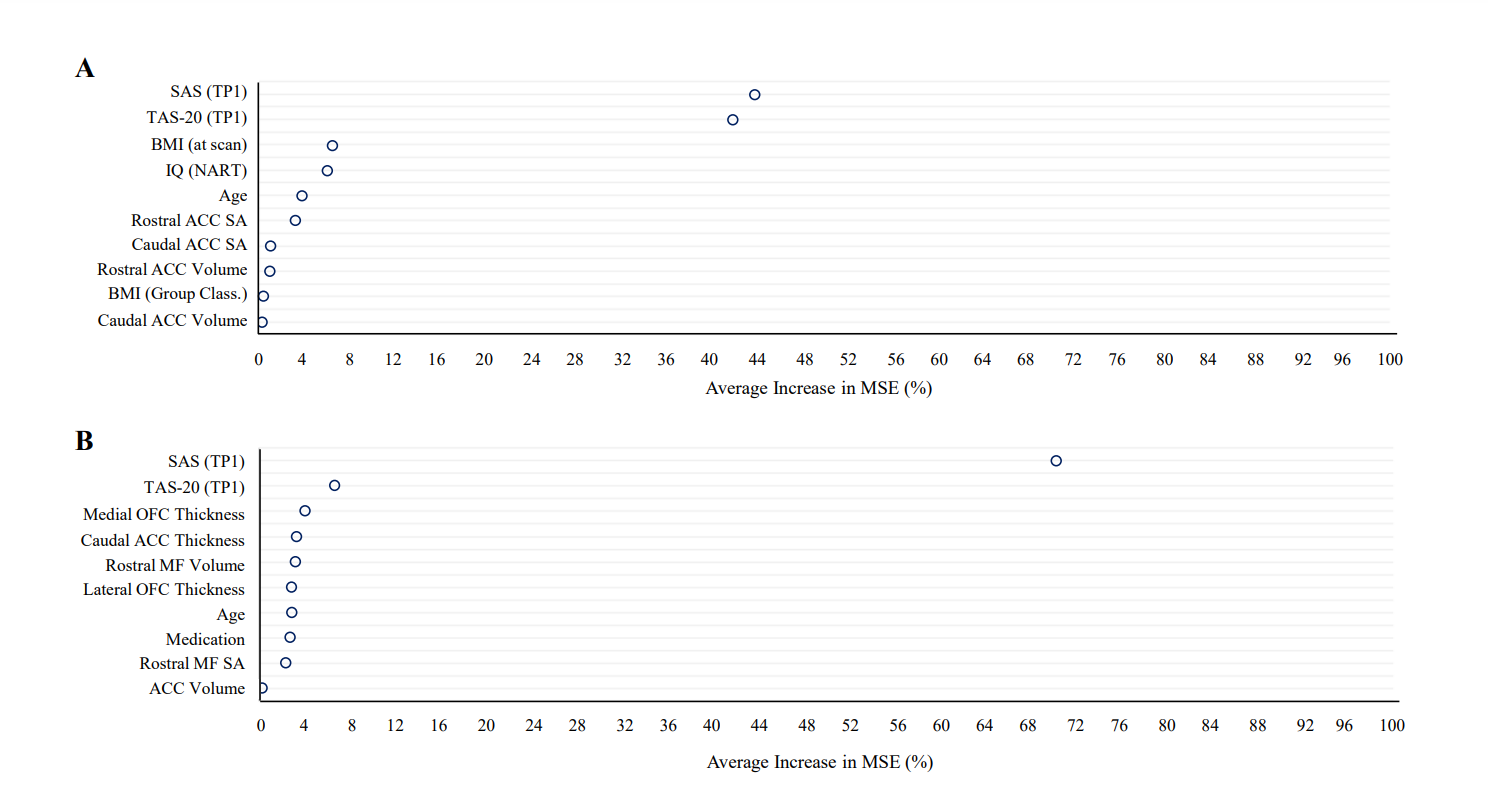
 **Supplementary Figure 3.** Importance rating of the top 10 variables used in a-priori RFR models to predict (**A**) TAS-20 scores and (**B**) SAS scores at research TP2.

[Abbreviations: ACC – Anterior Cingulate Cortex; AQ – Autism Quotient; BMI – Body Mass Index; Class. – Classification; IQ – Intelligence Quotient; MF – Middle Frontal Gyrus; MSE – Mean Squared Error; NART – National Adult Reading Test; OFC – Orbitofrontal Cortex; SAS – Social Anhedonia Scale; TAS – Toronto Alexithymia Scale; TP1 – Time-Point 1; TP2 – Time-Point 2]


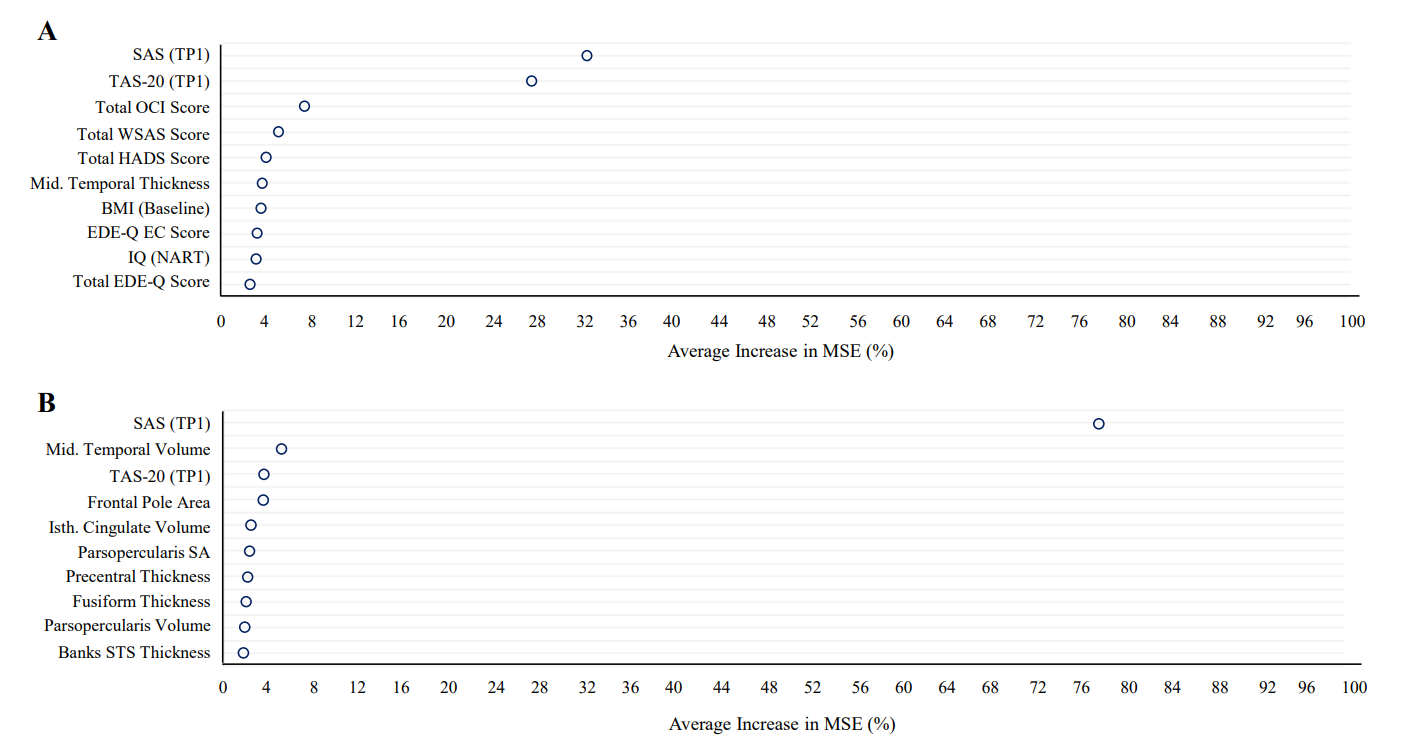


**Supplementary Figure 4.** Importance rating of the top 10 variables used in exploratory RFR models to predict (**A**) TAS-20 scores and (**B**) SAS scores at research TP2.

[Abbreviations: BMI – Body Mass Index; EC – Eating Concern; EDE-Q – Eating Disorder Examination Questionnaire; HADS – Hospital Anxiety and Depression Scale; Isth. – Isthmus; IQ – Intelligence Quotient; Mid. – Middle; MSE – Mean Squared Error; NART – National Adult Reading Test; OCI – Obsessive Compulsive Inventory; SA – Surface Area; SAS – Social Anhedonia Scale; STS – Superior Temporal Sulcus; TAS – Toronto Alexithymia Scale; TP1 – Time-Point 1; TP2 – Time-Point 2; WSAS – Work and Social Adjustment Scale]

**References**

1. Bebbington, P. E., & McManus, S. (2020). Revisiting the one in four: the prevalence of psychiatric disorder in the population of England 2000–2014. *The British Journal of Psychiatry*, *216*(1), 55-57.
2. First, M. B., Williams, J. B. W., Karg, R. S., & Spitzer, R. L. (2015). *Structured clinical interview for DSM-5—Research version (SCID-5 for DSM-5, research version; SCID-5-RV)*. Arlington, VA: American Psychiatric Association.
3. Halls, D., Leslie, M., Leppanen, J., Sedgewick, F., Surguladze, S., Fonville, L., ... & Tchanturia, K. (2021). The emotional face of anorexia nervosa: The neural correlates of emotional processing. *Human Brain Mapping*, *42*(10), 3077-3087.
